# Insights into the roles and driving forces of CCT3 in human tumors

**DOI:** 10.3389/fphar.2022.1005855

**Published:** 2022-10-12

**Authors:** Jingang Ma, Ping Song, Xinling Liu, Changgeng Ma, Mingzhu Zheng, Xiaomin Ren, Rui Wang, Wenshan Liu, Zhong Lu, Jiaqiu Li

**Affiliations:** ^1^ Department of Oncology, Affiliated Hospital of Weifang Medical University, School of Clinical Medicine, Weifang Medical University, Weifang, China; ^2^ Department of Gastroenterology, Affiliated Hangzhou First People’s Hospital, Medical School of Zhejiang University, Hangzhou, China; ^3^ Department of Hematology, Laboratory for Stem Cell and Regenerative Medicine, Clinical Research Center, Affiliated Hospital of Weifang Medical University, Weifang, China; ^4^ Department of Radiotherapy, Affiliated Hospital of Weifang Medical University, Weifang, China; ^5^ Shandong Key Laboratory of Clinical Applied Pharmacology, Department of Pharmacy, Affiliated Hospital of Weifang Medical University, Weifang, China

**Keywords:** CCT3, pan-cancer, biomarker, immune microenvironment, enhancers

## Abstract

CCT3 played a key role in many cancers. This study aimed to further explore the characteristics of CCT3 from a pan-cancer perspective and reveal the driving forces for CCT3. By bioinformatic analysis, we found that the mRNA and protein levels of CCT3 were abnormally elevated in most tumor types and were correlated with poor prognosis. Single-cell sequencing data indicated an abnormal increase of CCT3 expression in both malignant cells and multiple immune cells. In the tumor microenvironment, CCT3 expression was negatively relevant with immune cell infiltration and immune checkpoint genes expression. In colon cancer, knockdown of CCT3 inhibited cell proliferation. Gene set enrichment analysis showed that CCT3 may be oncogenic by regulating amino acid metabolism. Furthermore, we predicted sensitive drugs for CCT3 by virtual screening and sensitivity analysis. Many driver genes such as TP53 and KRAS were essential for CCT3 overexpression. Epigenetic factors, enhancers in particular, were also critical for CCT3 expression. Additionally, we constructed the lncRNA/circRNA-miRNA-CCT3 regulatory network. Collectively, CCT3 had the potential to be a diagnostic and prognostic biomarker for multiple tumor types. CCT3 expression was relevant with an immunosuppressive tumor microenvironment. CCT3 could be a new molecular target for colon cancer. Both genetic and epigenetic factors were responsible for CCT3 expression in tumors.

## Introduction

In 2020, the International Agency for Research on Cancer (IARC) reported that there were about 19.3 million new cancer cases and 10.2 million cancer deaths worldwide (Sung et al., 2021). Cancer remains one of the main diseases that cause human death worldwide. Despite the research on cancer has made great breakthroughs, finding new molecular therapeutic targets are still a requirement for tumor patients.

With the continuous development of many open bioinformatic databases such as TCGA (The Cancer Genome Atlas) and GEO (Gene Expression Omnibus) (Clough and Barrett, 2016; Blum et al., 2018), it has become an essential and important research method to use bioinformatics to explore medical questions. Chaperonin-containing TCP-1 (CCT) and HSP60 are the two main types of the chaperone system (Dong et al., 2020). CCT can promote the folding of a fraction of newly synthesized proteins such as KRAS, STAT3 and p53 (Liu et al., 2022a). CCT3 is one of the 8 subunits of CCT (Stoldt et al., 1996; Valpuesta et al., 2002; Macario and Conway de Macario, 2021) and has been explored its central roles in some tumors. For instance, CCT3 inhibitor inhibited the proliferation and migration in breast cancer (Xu et al., 2020). Abnormal expression of CCT3 significantly reduced the overall survival (OS) of hepatocellular carcinoma (HCC) patients (Cui et al., 2015). CCT3 knockdown blocked the proliferation of gastric cancer (GC) cells (Li et al., 2017). Meanwhile, the high expression of CCT3 also contributed to the progression of head and neck squamous cell carcinoma (HNSCC) (Wang et al., 2021). Interestingly, recent study showed that CCT3 is an RNA-binding protein (RBP) and could regulate lipid metabolism by LINC00326 in HCC (Sondergaard et al., 2022). In agreement with this study, CCT3 suppression led to oxidative stress and energy deficiency in breast and prostate cancer (Temiz et al., 2021). Moreover, CCT3 promotes cisplatin resistance by JAK2/STAT3 pathway in lung cancer (Danni et al., 2021). And CCT3 is responsible for castration-resistant prostate cancer (Lin et al., 2021a). Collectively, CCT3 plays a key role in cancer cell division, proliferation, metabolism and drug resistance. However, the roles of CCT3 in pan-cancer are not compared, especially from the immune aspect. And the upstream regulatory mechanisms for CCT3 overexpression in tumors are unclear.

In this study, a systematic pan-cancer analysis of CCT3 was performed by bioinformatics and experiments. We explored the characteristics of CCT3 in different tumors including expression levels, prognostic value and gene function. Meanwhile, we also analyzed the correlation between CCT3 expression and immune microenvironment. Furthermore, we predicted sensitive drugs for CCT3 by virtual screening and sensitivity analysis. Finally, the upstream regulatory mechanisms for CCT3 overexpression in tumors were elucidated. In summary, our results implied CCT3 as a potential biomarker for many tumors and CCT3 could be a new molecular target for colon cancer.

## Materials and methods

### The expression analysis of CCT3

The expression levels of CCT3 in normal tissues and tumor tissues were analyzed by SangerBox based on the Genotype Tissue Expression (GTEx) and the Cancer Cell Line Encyclopedia (CCLE) datasets. All abbreviations are showed in Supplementary Table S1. The TCGA and GTEx combination cohorts were used for statistical analysis by ACLBI database (https://www.aclbi.com/static/index.html#/). The gene expression RNA-seq data in 8 cancer types (BRCA, CHOL, COAD, LIHC, LUAD, LUSC, STAD and UCEC) were downloaded from UCSC Xena (https://xenabrowser.net/datapages/). The counts data were analyzed for upregulate differential genes using the R package ‘DESeq2’. In addition, we analyzed CCT3 expression differences between cancerous and adjacent tissues by TIMER, GEPIA2 and UANLCAN databases.

### Immunohistochemistry analysis of CCT3 protein

The Human Protein Atlas (HPA) (Karlsson et al., 2021) database provides pathological information on protein expression datasets from 17 different forms of human cancers. We downloaded IHC images of 5 tumor types-BRCA, LIHC, LUAD, LUSC and UCEC to identify the protein levels of CCT3 in cancerous and adjacent tissues.

### Analysis of the diagnostic value of CCT3

Gene Expression Profiling Interactive Analysis 2.0 (GEPIA2) (Li et al., 2021) is a web server that can be used for gene expression analysis. We explored the relevance between CCT3 expression and tumor clinicopathology through the “Stage plot” module. Besides, we calculated the relevance between CCT3 expression and tumor grades by using the TISIDB database (Ru et al., 2019).

### Prognostic value analysis of CCT3

The relevance between CCT3 expression and OS, DSS, DFI and PFI in 33 tumor types was analyzed by SangerBox. Based on the GEO, EGA, and TCGA datasets, survival data of CCT3 was evaluated by Kaplan-Meier Plotter (Lanczky and Gyorffy, 2021). In addition, we also calculated the correlation between the expression of CCT3 and overall survival by the “Survival Analysis” module of GEPIA2.

### Analysis of the single-cell sequencing data

The Tumor Immune Single Cell Center (TISCH) (Sun et al., 2021), the single cell RNA-seq database, can provide cell type annotation at the single-cell level for different cancer types. We obtained single-cell sequencing datasets through the TISCH database-BRCA (GSE114727), CHOL (GSE 125449), COAD (GSE146771), LIHC (GSE140228), NSCLC (GSE117570), STAD (GSE134520), UCEC (GSE154763), and quantified CCT3 expression levels by UMAP and violin plots.

### Analysis of the immunological features of CCT3

We explored the immune correlation using pan-cancer datasets from TCGA which integrates six algorithms including TIMER, xCell, MCP-counter, CIBERSORT, EPIC and quanTIseq. We calculated the relevance between CCT3 expression and immune cell infiltration by SangerBox. SIGLEC15, IDO1, CD274, HAVCR2, PDCD1, CTLA4, LAG3 and PDCD1LG2 are the immune checkpoint genes. We compared the relevance between CCT3 and these genes expression.

### Enrichment analysis of the CCT3-related genes

Through the STRING database (https://string-db.org/) (Szklarczyk et al., 2019), CCT3 was retrieved in the “Protein Names” module to get the CCT3 interaction proteins and the species were selected as homo species. We set the parameters as follows: the meaning of the network edge is “evidence”, the active interaction source is “Textmining” and “Experiment” and the minimum requirement interaction score is “high confidence (0.700)". The associated genes for CCT3 were obtained by the GENEMANIA database (Warde-Farley et al., 2010). The two groups of genes were combined for GO and KEGG enrichment analysis by DAVID database (Sherman et al., 2022).

### Analysis of genetic and epigenetic alteration for CCT3

The mutational characteristics of CCT3, including mutation type and frequency, were explored through the cBioPortal database (Cerami et al., 2012). The effect of driver genes on CCT3 expression in cancers was showed by the TCGA portal database (Xu et al., 2019). The effect of the TP53 mutation on the CCT3 expression in the tumors was analyzed by the UALCAN database. The CpG islands in the CCT3 promoter region were observed by Methprimer database (Li and Dahiya, 2002). And the effect of DNA methylation on CCT3 expression in the pan-cancer was analyzed by DiseaseMeth database (Xiong et al., 2017). Finally, we analyzed the enrichment of H3K27ac signals in CCT3 gene loci by WashU Epigenome Browser (http://epigenomegateway.wustl.edu/browser/).

### Sensitive drugs and molecular docking

We obtained RNA expression and drug data for NCI-60 cell lines from the CELLMiner database (Reinhold et al., 2012). CCT3 expression and drug sensitivity (IC50) was calculated and visualized by R software. The spatial structure of the CCT3 protein was obtained from the Protein Data Bank (PDB) and the drug molecular structure was obtained from the Pubchem database. The GHECOM algorithm was used to identify the CCT3 protein-binding sites. The UCSF DOCK 6.9 software was used for molecular docking. And it was visualized by PyMol. Finally, Ligplus was used to analyze the interaction forces.

### CeRNA regulatory network analysis

The miRNAs targetingCCT3 were predicted by 5 databases including StarBase (Li et al., 2014), Targetsacn (McGeary et al., 2019), MiRDB (Chen and Wang, 2020), MiRWalk (Dweep and Gretz, 2015) and DIANA (Paraskevopoulou et al., 2013). Then we compared and obtained the intersection. Then, the upstream lncRNAs/circRNAs were predicted by StarBase database. Finally, the lncRNA/circRNA-miRNA-CCT3 regulatory network was visualized by Cytoscape software.

### Cells

All cells were obtained from cell bank of the Chinese Academy of Sciences. HCT116 was cultured in 10 cm dish containing McCoy’s 5A medium supplemented with 10% FBS. DLD1, SGC7901 and BGC823 were cultured in 10 cm dish containing RPMI 1640 medium supplemented with 10% FBS. The culture conditions were 37°C, 5% CO2 and 95% humidity.

### Cell transfection

1 × 10^5^cells in 6-well plates were transfected with siRNA by RNAiMAX (Invitrogen, 13778150) or added JQ1-1uM and I-BET-762-2uM (Selleck, S7110 and S7189) for 24h. The siRNA was purchased from Gene Pharma (Shanghai, China). The siRNA sequences used were listed below:

CCT3-siRNA-1#: S: GCU​GUG​AAG​CUG​CAG​ACU​UTT, AS: AAG​UCU​GCA​GCU​UCA​CAG​CTT; CCT3-siRNA-2#: S: GCA​AGG​CAU​UGG​AUG​AUA​UTT, AS: AUA​UCA​UCC​AAU​GCC​UUG​CTT; BRD4-siRNA-1#: S: CCG​UGA​UGC​UCA​GGA​GUU​UTT, AS: AAA​CUC​CUG​AGC​AUC​ACG​GTT; BRD4-siRNA-2#: S: AGC​UGA​ACC​UCC​CUG​AUU​ATT, AS: UAA​UCA​GGG​AGG​UUC​AGC​UTT.

### Quantitative real-time PCR

The qPCR assays were performed as reported previously (Li et al., 2018). Briefly, the extraction of total RNA from cells was performed by Trizol following the manufacturer’s instructions. And after RNA quantification, cDNA was synthesized. Next, mRNA expression was detected by qPCR using UltraSYBR Mixture (CW0957M, cwbiotech). The primer sequences used were listed below:

CCT3-F: TTT​GGA​CCC​AAT​GGG​AGG​C, R: ACA​GCA​TTT​CCC​CTG​CAA​GAA​T; Tubulin-F: GAA​GCA​GCA​ACC​ATG​CGT​GA, R: AAG​GAA​TCA​TCT​CCT​CCC​CCA.

### CCK-8 assay

3 × 10^3^ cells were seeded in the 96-well plate and were incubated in an incubator for 72 h 10ul CCK8 reagent (Beyotime, C0038) and 100ul culture medium were then added to each well and incubated for 30min. Finally, the absorbance at 490 nm in each well was measured using a microplate reader.

### Colony formation assay

1 × 10^5^ cells were uniformly spread in 6-well plates overnight and then were treated for CCT3 knockdown with the corresponding siRNA. After 3 days, 1000 cells per well were reseeded in 6-well plates and cultured for 2 weeks. Then the medium was taken away and the cells were washed with PBS to remove impurities. Cells were fixed with paraformaldehyde (4% concentration, 20 min, room temperature). Finally, cells were stained with 0.1% crystal violet solution and then counted.

### Chromatin immunoprecipitation

The ChIP assay was performed as reported previously (Song et al., 2020). Anti-H3K27ac antibody was purchased from Abcam (ab177178). Anti-BRD4 antibody was purchased from Bethyl Laboratories (A301-985A100). The purified DNA was analyzed by qPCR. The primer sequences used were listed below: CCT3-H3K27ac-CHIP-F: GCC​TCT​CTA​GTC​CAC​CTG​TTG, R: ACT​GTG​TAT​TGC​GAC​TCG​GC.

### Statistical analysis

Two-sided student’s t-test was used to assess statistical significance by using GraphPad Prism seven software and the results were presented as mean ± SD. All the experiments were repeated in triplicate. If the results do not have the same SD, then we used *t*-test with Welch’s correction. The *p* value < 0.05 was considered statistically significant. *p < 0.05; **p < 0.01; ***p < 0.001; ****p < 0.0001.

## Results

### CCT3 expression in pan-cancer

**FIGURE 1:**
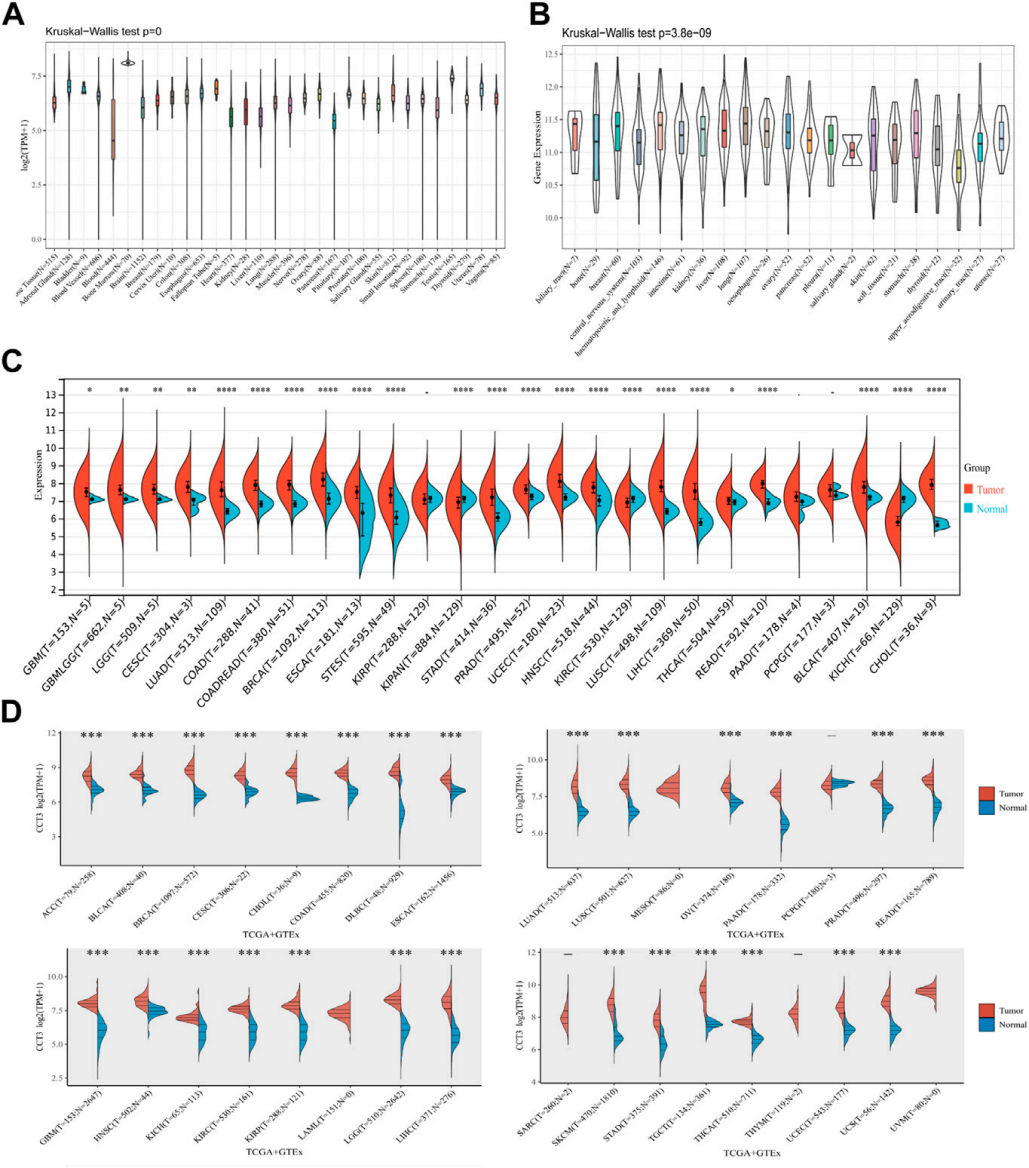
Expression levels of CCT3 in pan-cancer. **(A)** The expression levels of CCT3 in normal tissues were analyzed using the GTEx datasets. **(B)** The expression levels of CCT3 in different tumor tissues were analyzed using the CCLE datasets. **(C)** Differential expression levels of CCT3 in tumor and corresponding normal tissues were analyzed using the TCGA datasets. **(D)** Differential expression levels of CCT3 in tumor and corresponding normal tissues were analyzed using the TCGA and GETx datasets. *p < 0.05, **p < 0.01, ***p < 0.001.

**FIGURE 2:**
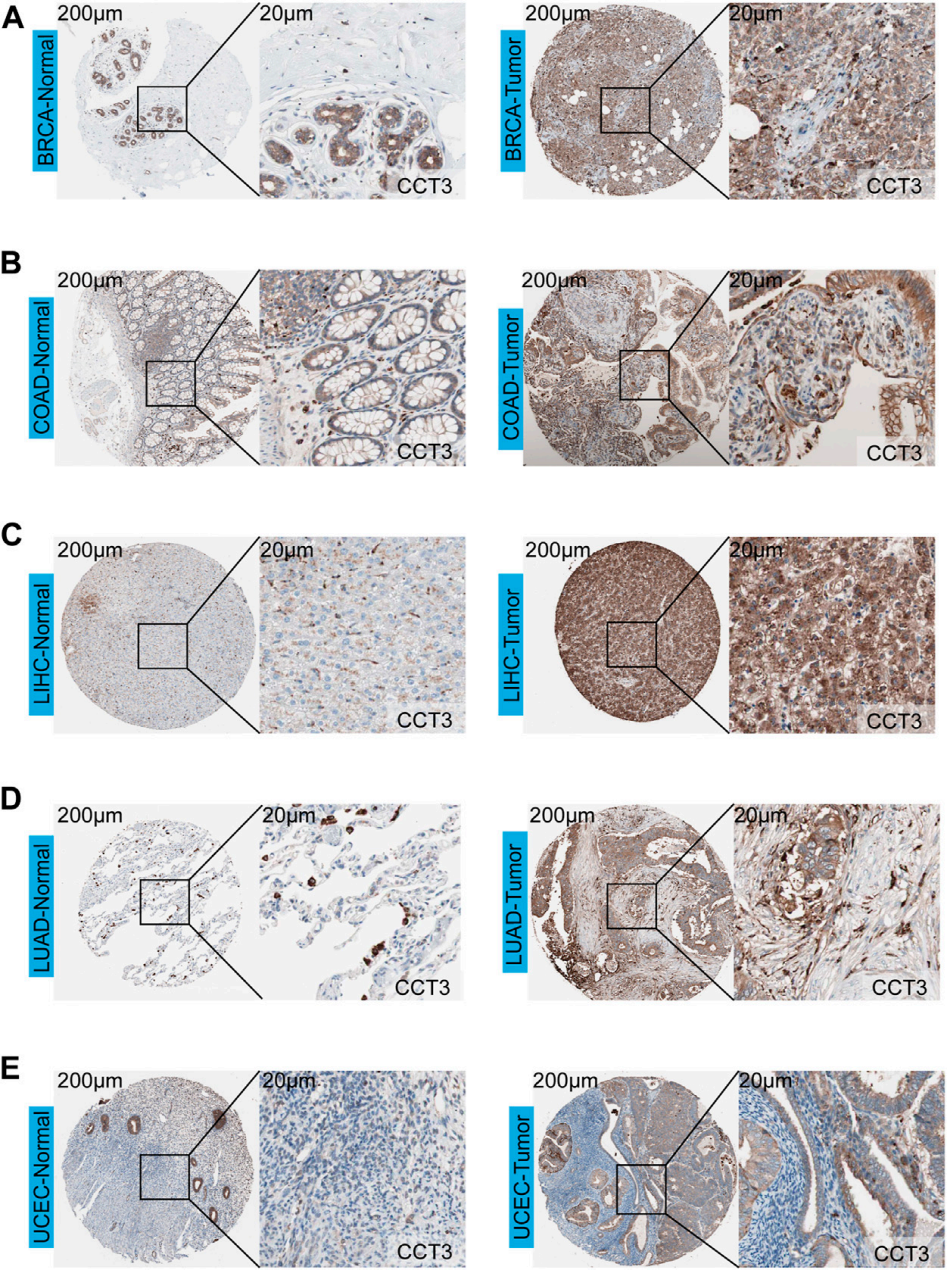
Immunohistochemistry analysis of CCT3 protein. The protein levels of CCT3 were analyzed by IHC in several cancer types: **(A)** BRCA, **(B)** COAD, **(C)** LIHC, **(D)** LUAD, **(E)** UCEC.

First, we analyzed the mutation status of CCT3 in tumors by the cBioportal database (Supplemenrtary Figure S1A,B). The results showed the mutation frequency of CCT3 was rare in pan-cancer. To obtain a detailed understanding of CCT3 expression in normal and tumor tissues, CCT3-related expression was specifically analyzed by SangerBox. First, we calculated the expression levels of CCT3 in normal tissues by analyzing the GTEx datasets (Figure 1A). Next, we calculated CCT3 expression levels in different tumor tissues by analyzing the CCLE datasets (Figure 1B). Moreover, based on the TCGA datasets, we analyzed the CCT3 differential expression levels between carcinomas and adjacent tissues (Figure 1C). The results showed that CCT3 expression was up-regulated in 20 cancer types and down-regulated in three cancer types. To take a closer step into the aberrant expression of CCT3 in pan-cancer, we analyzed CCT3 expression using a combined cohort of TCGA and GTEx in ACLBI. In the 33 cancer types, we found abnormal high expression of CCT3 in 27 of these cancer types (Figure 1D). In addition, 8 cancer types including BRCA, CHOL, COAD, LIHC, LUAD, LUSC, STAD and UCEC, showed abnormal high expression of CCT3 in other three databases-TIEMR, GEPIA and UALCAN (Supplementary Figure S2A–C). Then we calculated the ranking of CCT3 expression among the differentially expressed genes in the 8 high-expressed cancer types (Supplementary Table S1). Notably, in BRCA, COAD, LIHC, LUAD and LUSC, CCT3 belongs to the top 10% differentially expressed genes. Besides, we found a close correlation between CCT3 expression and pathological stages of multiple cancer types, including KICH, KIRP, LIHC, LUAD, LUSC and STAD (Supplementary Figure S3A). And we observed a close link between CCT3 expression and tumor grades of multiple cancer types, including CESC, KIRC, LIHC, STAD and UCEC (Supplementary Figure S3B). Finally, we explored the protein expression levels of CCT3 in the above 8 tumors by immunohistochemistry analysis and found that the protein expression levels of CCT3 were obvious higher in BRCA, COAD, LIHC, LUAD and UCEC than the corresponding normal tissues (Figures 2A–E). In summary, the above results imply a potential of CCT3 as a diagnostic biomarker for many tumor types.

### Prognostic value of CCT3

**FIGURE 3:**
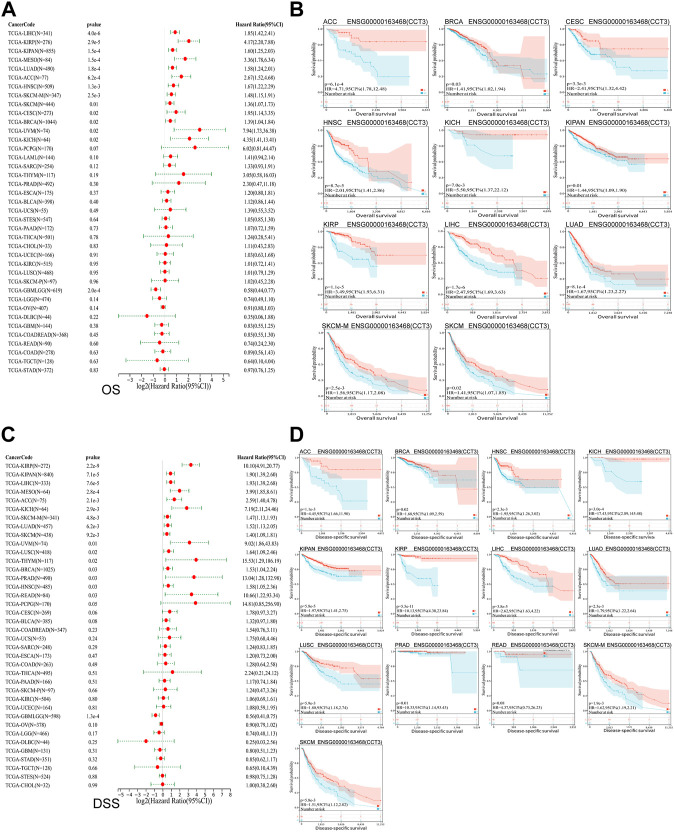
Prognostic value of CCT3 for overall survival (OS) and disease-specific survival (DSS) in pan-cancer. **(A)** Forest plots showed the correlation between CCT3 expression and OS in 33 cancer types. **(B)** The KM curves showed the correlation between CCT3 expression and OS in pan-cancer. **(C)** Forest plots showed the correlation between CCT3 expression and DSS in 33 cancer types. **(D)** The KM curves showed the correlation between CCT3 expression and DSS in pan-cancer.

**FIGURE 4:**
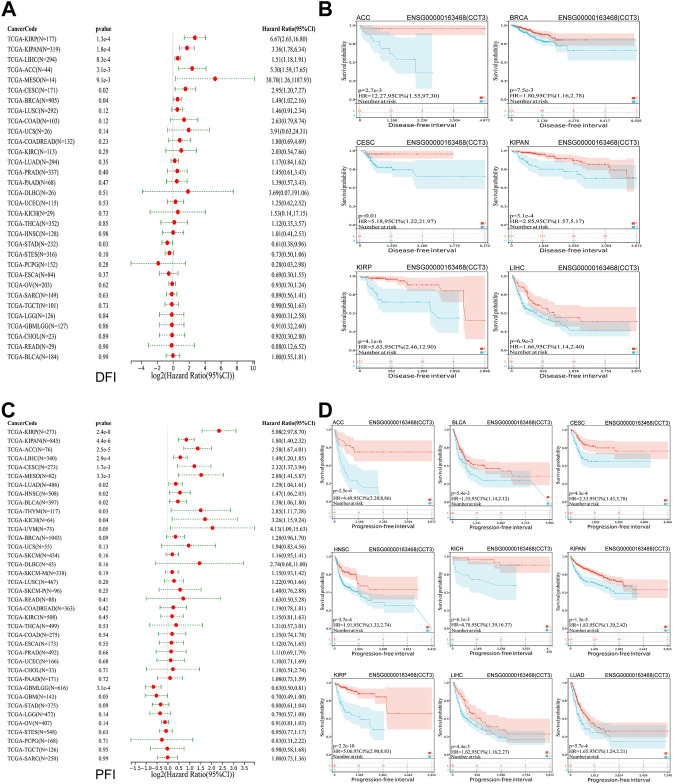
Prognostic value of CCT3 for disease-free interval (DFI) and progression-free interval (PFI) in pan-cancer. **(A)** Forest plots showed the correlation between CCT3 expression and DFI in 33 cancer types. **(B)** The KM curves showed the correlation between CCT3 expression and DFI in pan-cancer. **(C)** Forest plots showed the correlation between CCT3 expression and PFI in 33 cancer types. **(D)** The KM curves showed the correlation between CCT3 expression and PFI in pan-cancer.

Whether the high-expressed CCT3 has a key prognostic value in tumors? Then we calculated the relevance between CCT3 expression and the prognosis in 33 cancer types. The prognosis contains overall survival (OS), disease-specific survival (DSS), disease-free interval (DFI) and progression-free interval (PFI). The forest plot showed that CCT3 acted as a risk factor for OS in 12 cancer types (Figure 3A). In addition, the KM curves showed that high CCT3 expression was associated with poor OS in 11 cancer types (Figure 3B). And CCT3 expression was significantly relevant with DSS in 14 tumors (Figure 3C). Among them, the high expression of CCT3 resulted in poor DSS in 13 tumors (Figure 3D). Meanwhile, abnormal high expression of CCT3 was related with DFI and PFI in six cancers and 9cancers, respectively (Figures 4A–D). Furthermore, based on TCGA or GEO datasets, we found a negative correlation between CCT3 high expression and the OS of multiple tumor types by Kaplan-Meier Plotter and GEPIA2 databases (Supplementary Figure S4A,B). All the data suggest CCT3 might be a prognostic biomarker for a number of tumor types.

### Immune characteristics of CCT3

**FIGURE 5:**
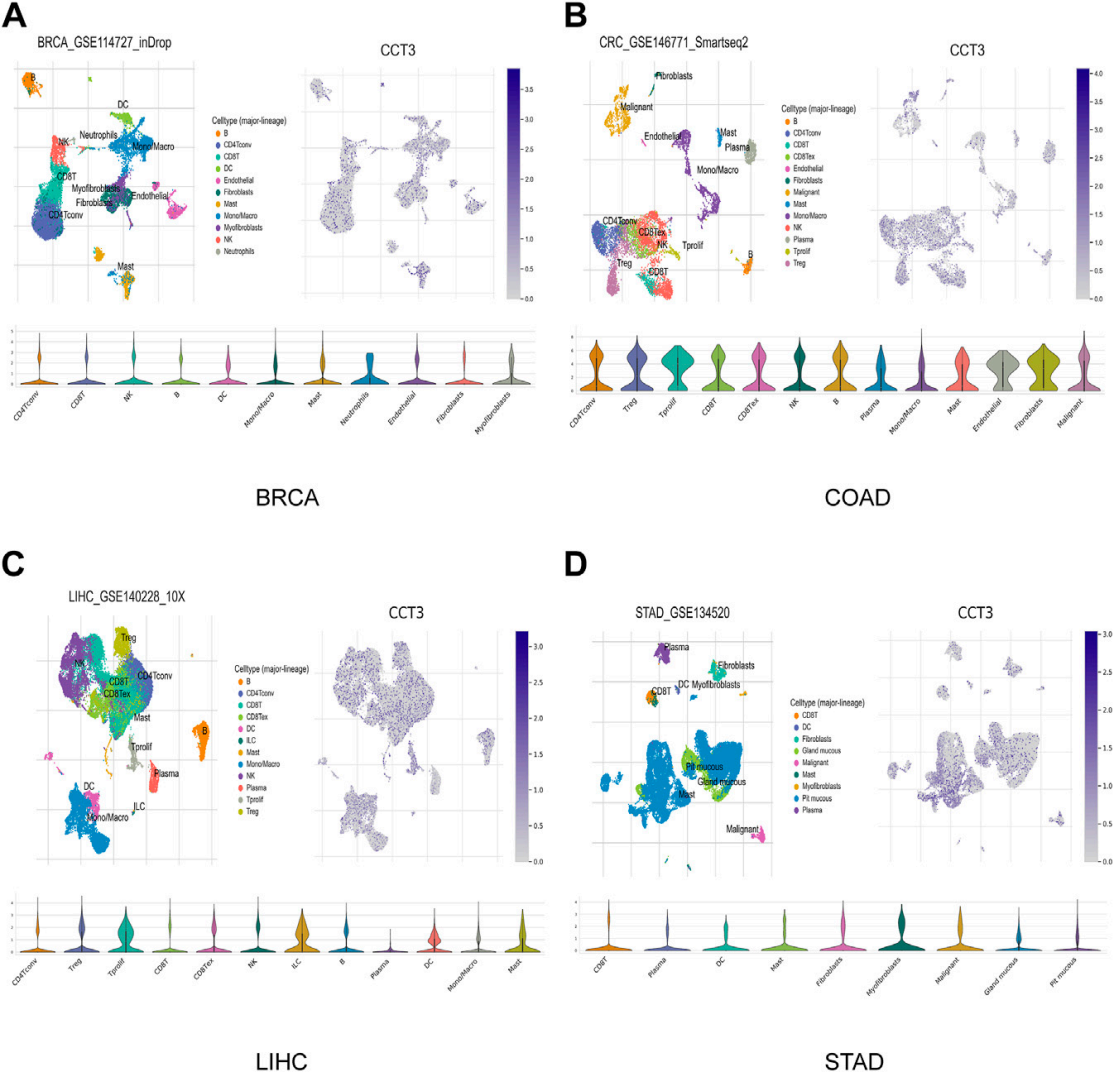
CCT3 expression based on the single-cell sequencing data. CCT3 expression in various cell types was investigated by single-cell sequencing data in **(A)** BRCA, **(B)** COAD, **(C)** LIHC and **(D)** STAD.

**FIGURE 6:**
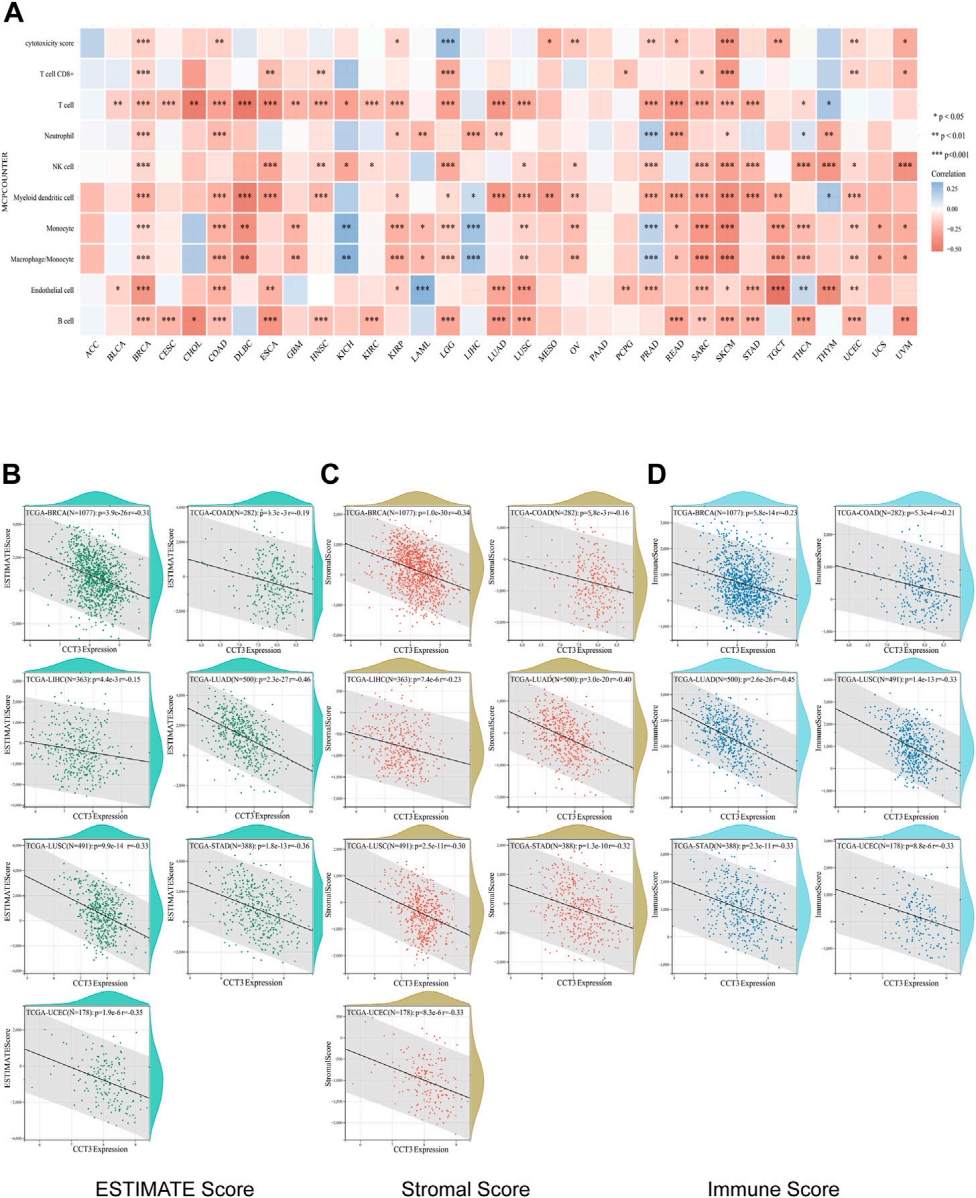
Correlation between CCT3 expression and immune cell infiltration. **(A)** Correlation between CCT3 expression and immune cell infiltration was analyzed using the MCP-counter algorithm in the R package “immunedeconv”. **(B–D)** Estimate Immune-infiltration score in 8 tumors (BRCA, CHOL, COAD, LIHC, LUAD, LUSC, STAD and UCEC) based on CCT3 expression using the R package ‘ESTIMATE'.

**FIGURE 7:**
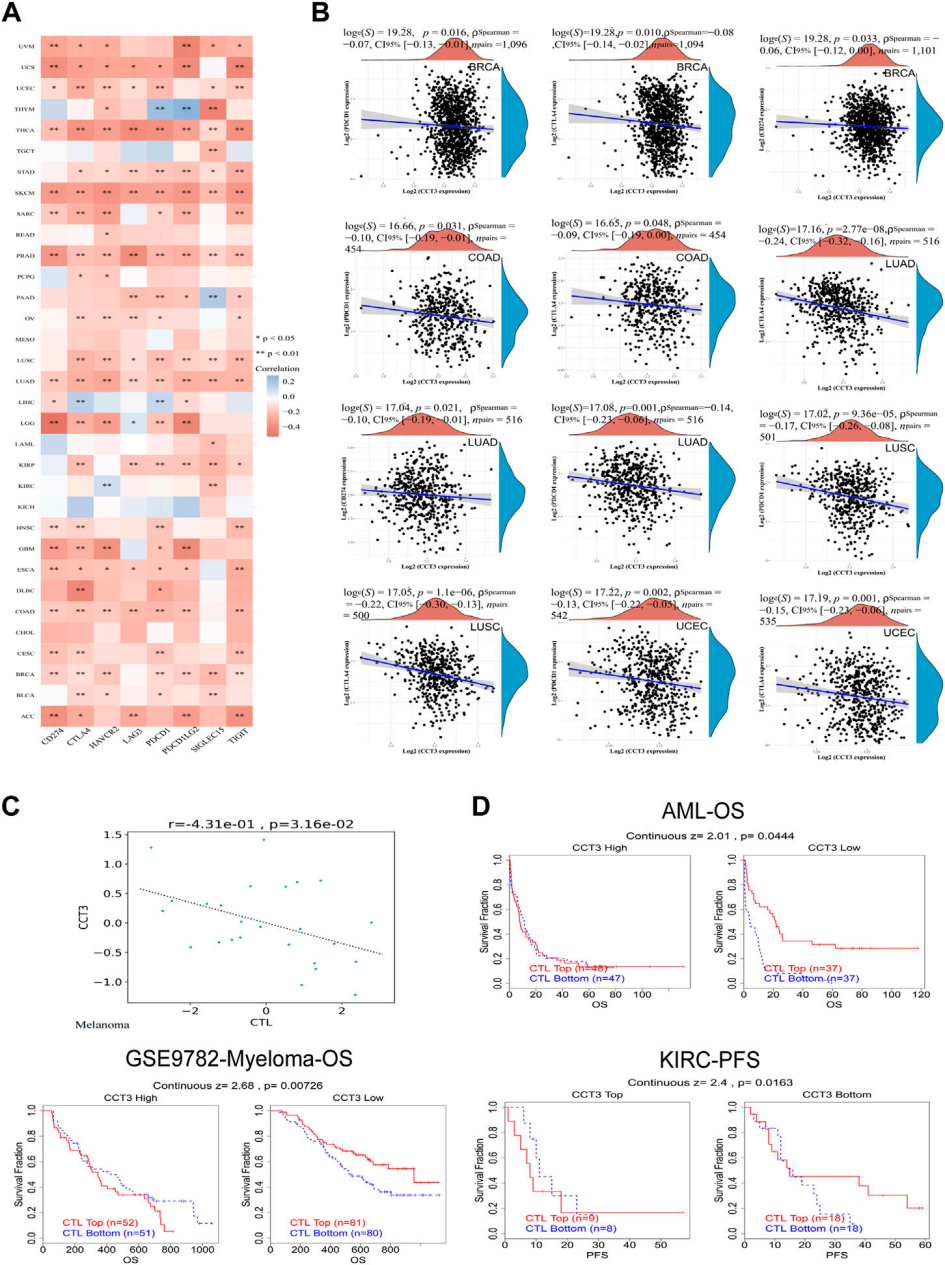
Correlation between CCT3 and immune checkpoint gene expression. **(A)** Heatmap showed the correlation between CCT3 and immune checkpoint gene expression in pan-cancer. **(B)** The correlation between CCT3 and CD274, CTLA4 and PDCD1 expression in ACLBI database. **(C)** The correlation between CCT3 and cytotoxic T lymphocyte (CTL). **(D)** The prognosis of tumor patients with different CCT3 expression and CTL levels. *p < 0.05, **p < 0.01, ***p < 0.001.

Single-cell sequencing was used to reveal the specific characteristics of genes in cancer cells, as well as in tumor microenvironment (Zhang et al., 2022). Interestingly, among the CCT3 high-expressed 8 tumors, other than in malignant cells, we also found an abnormal increase of CCT3 expression in multiple immune cells of the tumor microenvironment in CHOL, COAD, NSCLC and STAD (Figure 5 and Supplementary Figure S5). Particularly, the highest cancer type of CCT3 expression in the tumor microenvironment was COAD. As is known to all, the immune cells in tumor microenvironment play a central part in the immunotherapy (Uyanik et al., 2021). Therefore, we wondered the relevance between CCT3 expression and immune characteristics. We discovered that CCT3 expression was negatively relevant with immune cell infiltration in most of tumor types (Figure 6A and Supplementary Figure S6). Moreover, our findings revealed that CCT3 expression was negatively relevant with immune scores including ESTIMATE Score, Stromal Score and Immune Score (Figures 6B–D). Besides, we identified that the immune checkpoint genes expression including CD274, PDCD1 and CTLA4, was negatively relevant withCCT3 expression (Figures 7A,B). Interestingly, we found that CCT3 expression was negatively relevant with the roles of cytotoxic T lymphocyte (CTL) (Figures 7C,D). In conclusion, CCT3 expression was relevant with immunosuppressive tumor microenvironment. And CCT3 may be a novel target of immunotherapy.

### Functional enrichment analysis of CCT3

**FIGURE 8:**
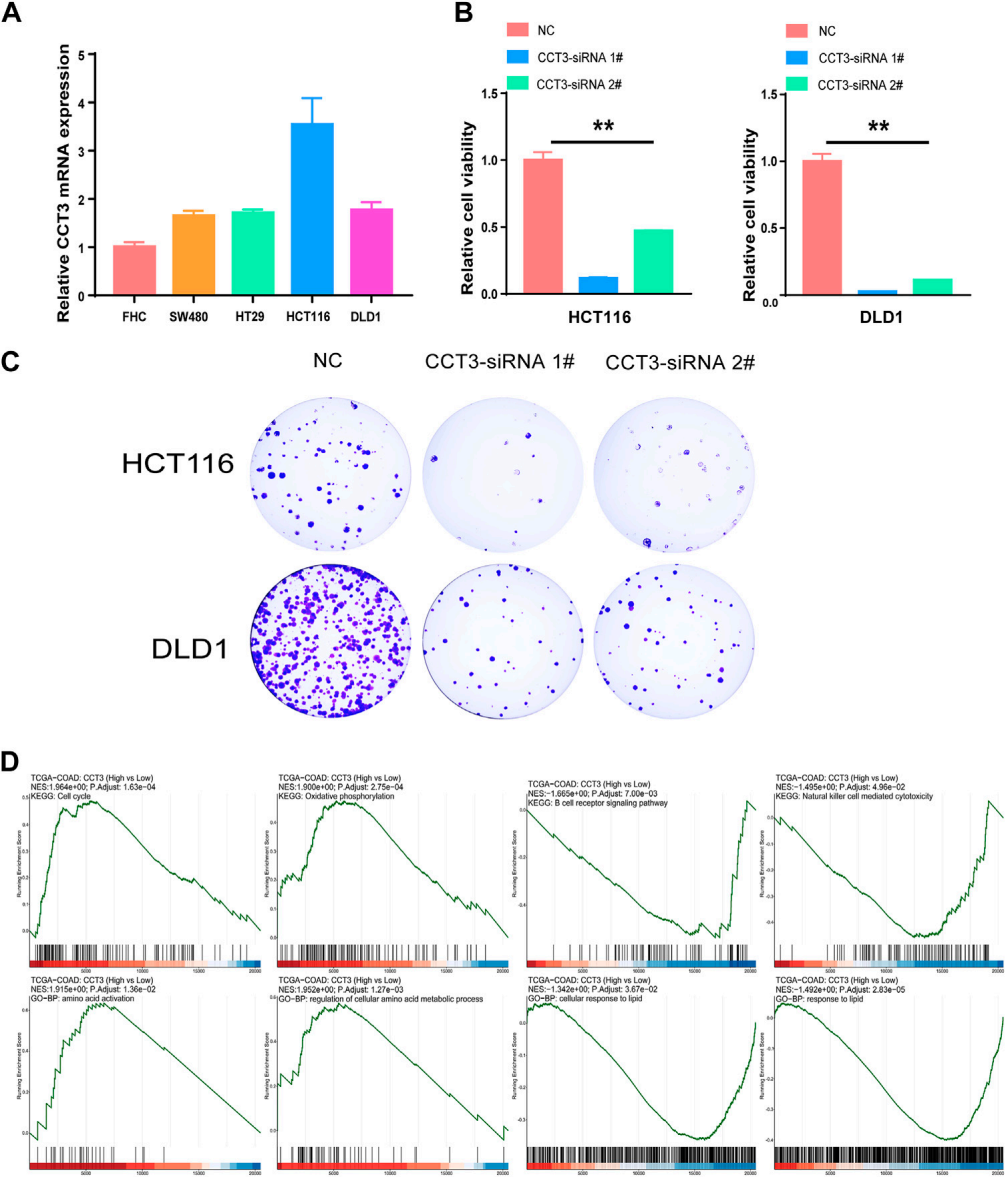
The effect of targeting CCT3 in COAD. **(A)** The expression levels of CCT3 in COAD cell lines were detected by qPCR assay. **(B)** The effect of CCT3 knockdown on the cell viability was determined by CCK-8 assay. **(C)** Colony formation assay was conducted after CCT3 knockdown. **(D)** Gene set enrichment analysis (GSEA) for CCT3 in COAD.

**FIGURE 9:**
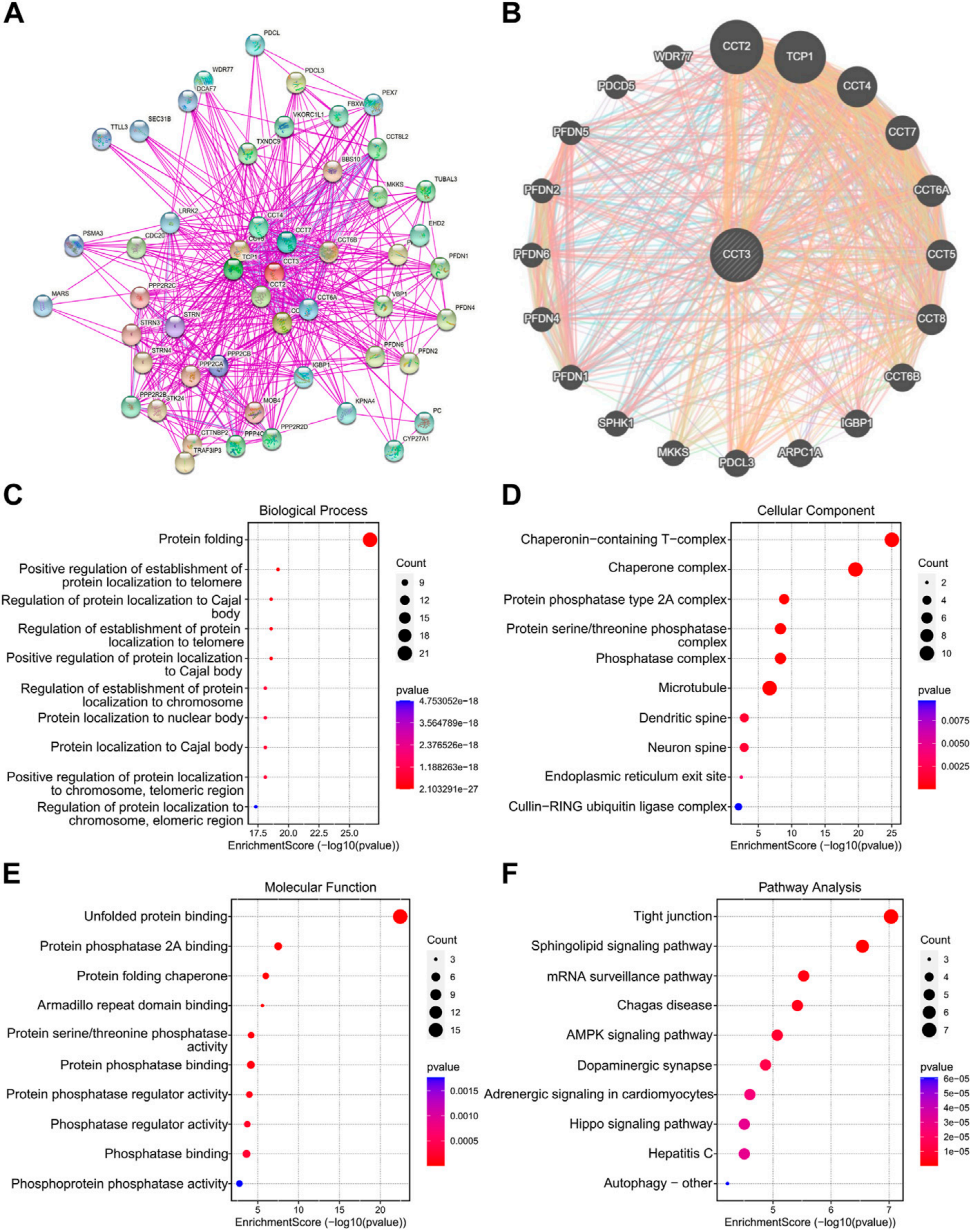
Functional enrichment analysis of CCT3. **(A)** The protein-protein interaction (PPI) networks for CCT3 were obtained by the STRING tool. **(B)** The top 20 CCT3-related genes were displayed using the GeneMANIA tool. **(C–F)** GO and KEGG analysis for CCT3-related genes. *p < 0.05, **p < 0.01, ***p < 0.001.

Considering that CCT3 showed higher expression levels in both cancer cells and tumor microenvironment in COAD and STAD than other tumors, we wondered whether targeting CCT3 was feasible in gastrointestinal tumor. Previous study suggested that CCT3 was critical for gastric cancer cell growth (Li et al., 2017). Now we explored CCT3’s function in colon cancer cells. QPCR assay validated that CCT3 mRNA levels in the colon normal cell line-FHC was lower than those in the COAD tumor cell lines (Figure 8A). Knockdown of CCT3 expression in the COAD cell lines inhibited the cell viability and colony formation ability (Figures 8B,C). Next, gene set enrichment analysis (GSEA) showed that amino acid active, regulation of cellular amino acid metabolic process, oxidative phosphorylation and cell cycle were mainly enriched in the CCT3 high-expressed group. However, response to lipid, positive regulation of lipid localization, natural killer cell mediated cytotoxicity and B cell receptor signaling pathway were enriched in the CCT3 low-expressed group (Figure 8D). This is consistent with the above findings that CCT3 expression was negative relevant with tumor immune process. To further explore the functions of CCT3 in cancers, we obtained the CCT3-related genes through the online analysis tools String and GENEMANIA (Figures 9A,B). Then we performed GO and KEGG enrichment analysis for these genes. The results showed that the main functional enrichment of biological processes (BP) is protein folding (Figure 9C). The cellular component (CC) is mainly chaperonin−containing T−complex (Figure 9D). Molecular function (MF) is mainly involved in the unfolded protein binding (Figure 9E). KEGG enrichment analysis revealed that CCT3-related genes were enriched in Sphingolipid signaling pathway, mRNA surveillance pathway, AMPK signaling pathway and Hippo signaling pathway (Figure 9F). In summary, our results revealed the important functions of CCT3 in tumorigenesis.

### Predicting sensitive drugs for CCT3 protein

**FIGURE 10:**
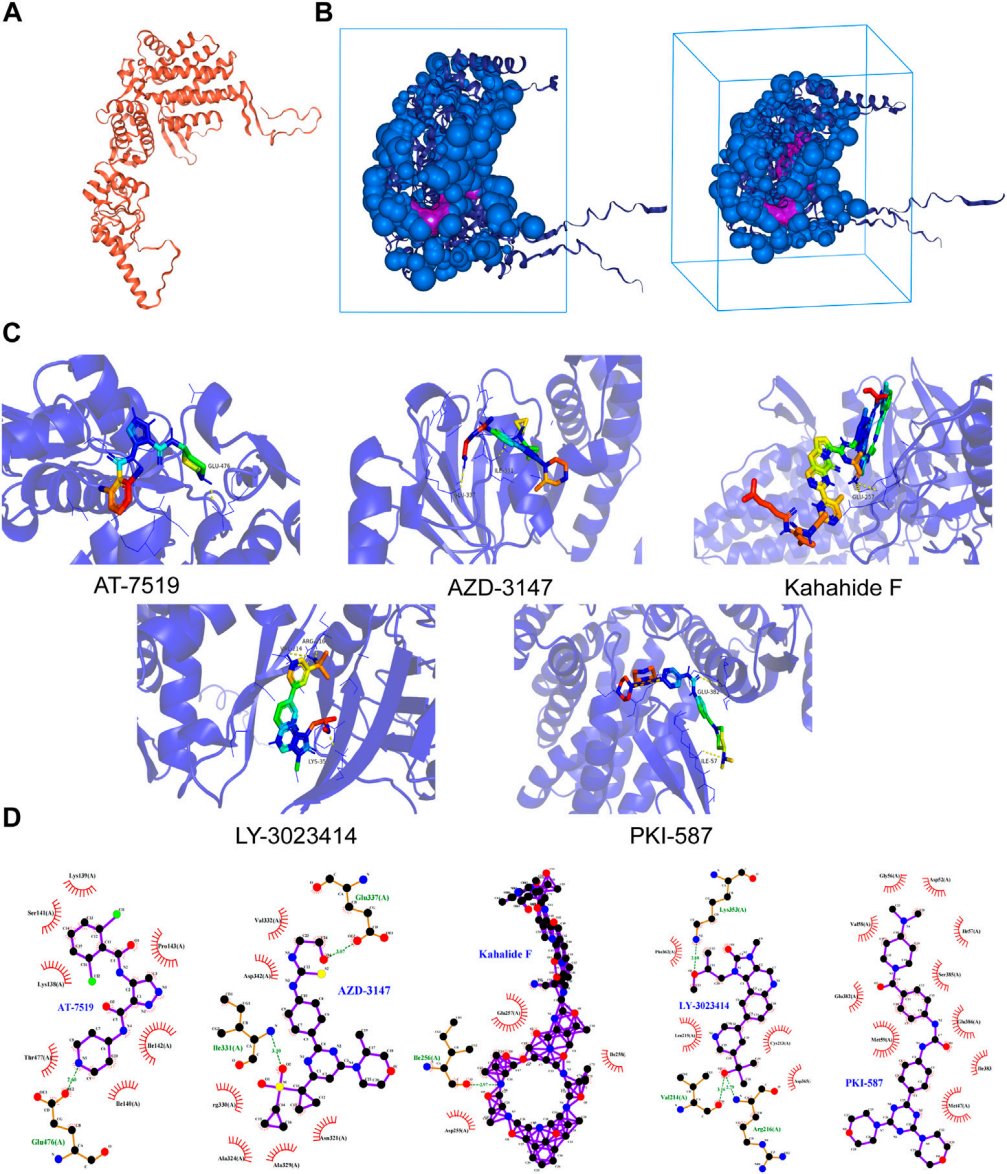
Predicting sensitive drugs for CCT3 protein. **(A)** Three-dimensional structure of CCT3 protein. **(B)** Predicted binding sites and box of CCT3 protein. **(C)** The interaction between sensitive drugs and CCT3 protein by PyMol software. **(D)** The two-dimensional structure of the interaction force between the compound (middle) and the amino acid residues of CCT3 protein (green dotted lines represents the hydrogen bonds).

Subsequently, we wanted to predict sensitive drugs for targeting CCT3 protein. First, the FDA-approved clinical trial drugs were screened through the CellMiner database to obtain the CCT3-related susceptible drugs (Supplementary Figure S7A,B). Among them, A1210477, LY-3023414, PKI-587, AT-7519, Kahalide F and AZD-3147 were screened out according to the correlation. Meanwhile, the spatial structure of the CCT3 protein was obtained by the PDB database (Figure 10A). The binding sites and boxes for the CCT3 protein were obtained by the GHECOM algorithm (Figure 10B). Compound structures of the six drugs were obtained through the PubChem database (Supplementary Figure S7C). Next, we used AutoDock for molecular docking and obtained the free binding energies (Supplementary Table S2). We used the PyMol software to visualize the interaction between 5 sensitive drugs and CCT3 protein (Figure 10C). Finally, we calculated the interaction force of the docking conformation by Ligplus (Figure 10D). In conclusion, we predicted several sensitive drugs for CCT3 protein.

### The effect of genetic and epigenetic factors on CCT3 expression

**FIGURE 11:**
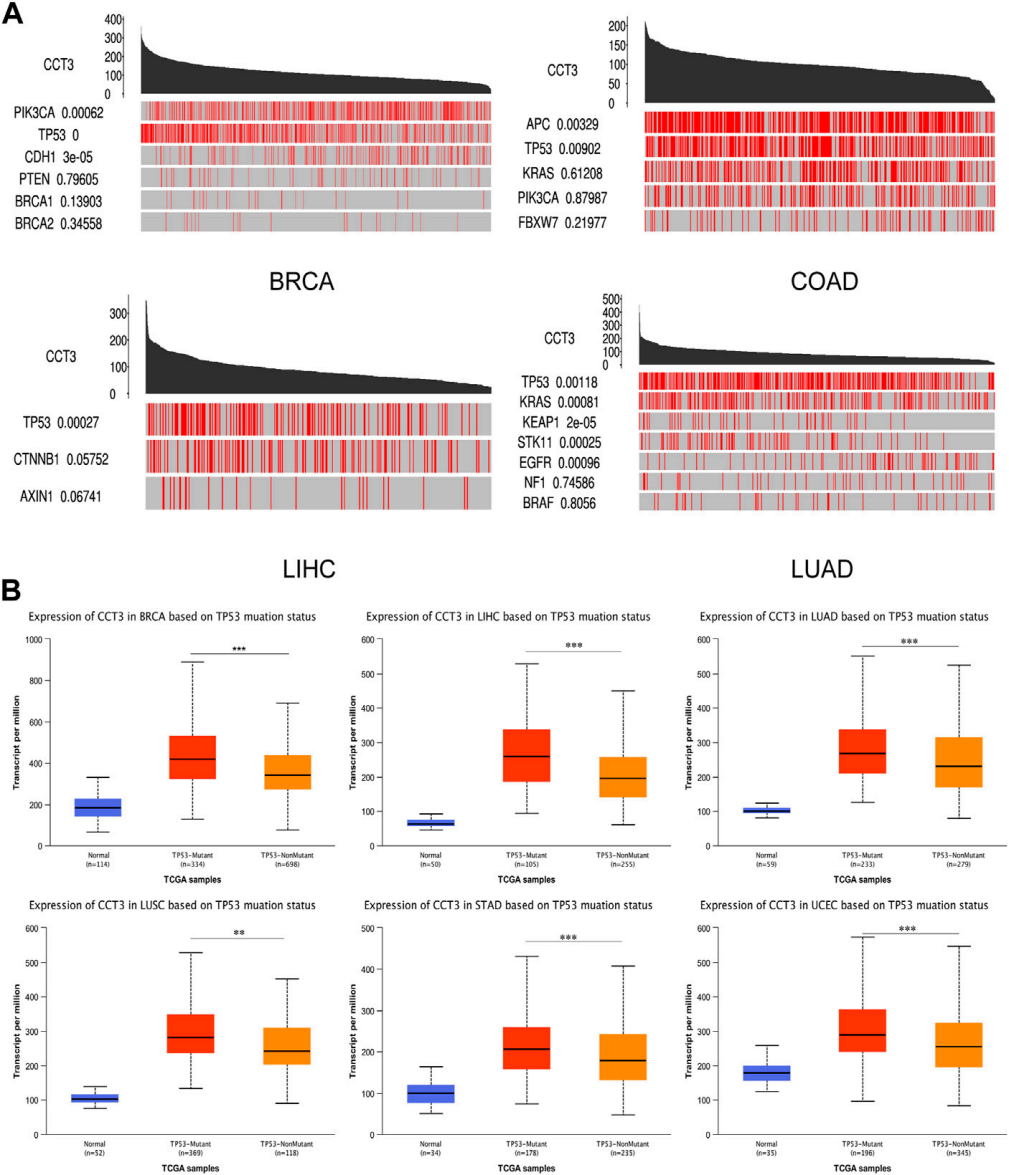
The effect of driver genes on CCT3 expression. **(A)** The correlation between CCT3 expression and driver genes in four cancers (BRCA, COAD, LIHC, LUAD) was analyzed using the TCGA portal database. **(B)** The correlation between CCT3 expression and TP53 mutation status was analyzed using the UALCAN database. *p < 0.05, **p < 0.01, ***p < 0.001.

**FIGURE 12:**
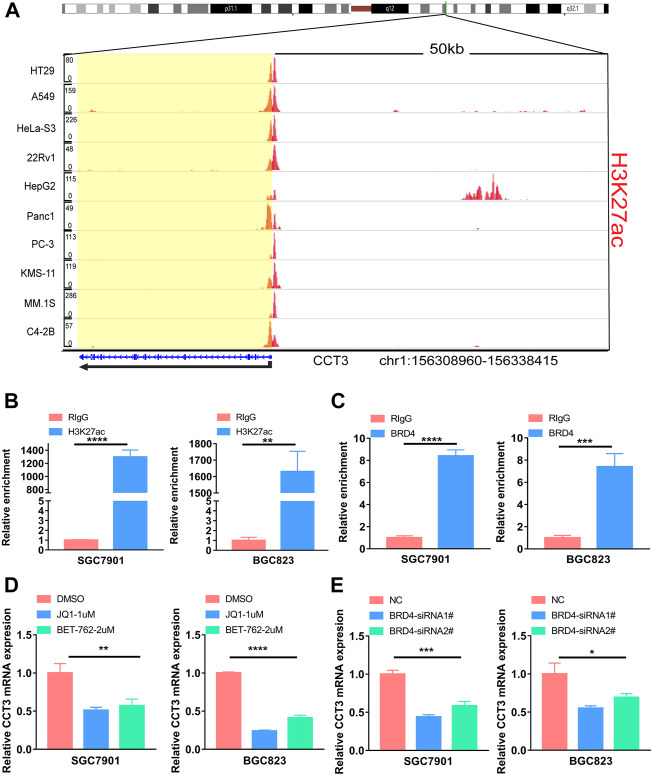
The effect of enhancers on CCT3 expression. **(A)** The H3K27ac signal of the CCT3 gene loci in different tumor cell lines was showed by WashU Epigenome Browser. **(B–C)** The relative enrichment levels of H3K27ac or BRD4 to CCT3 gene loci by ChIP-qPCR in gastric cancer cells. **(D–E)** The relative CCT3 expression after the treatment with enhancer inhibitors (JQ1 and I-BET-762) or BRD4 siRNAs.

**FIGURE 13:**
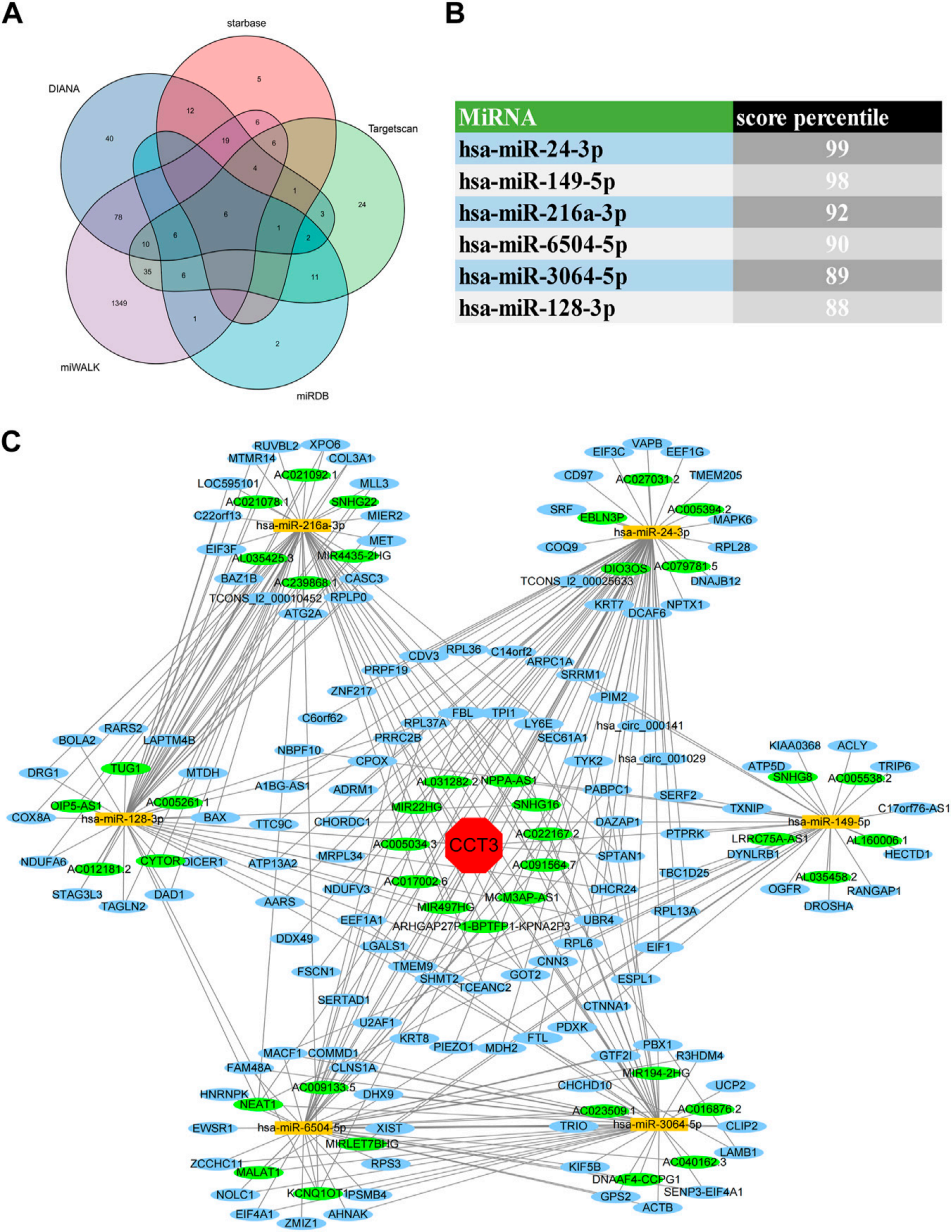
CeRNA regulatory network analysis for CCT3. **(A)** The Venn diagrams showed the predicted miRNAs complementary to CCT3 3′UTR region. **(B)** Targetscan database showed the score percentile of the predicted six miRNAs. **(C)** The ceRNA regulatory network for CCT3 was constructed by Cytoscape.

For better targeting CCT3 to treat tumors, it should reveal the mechanisms for the abnormal expression of CCT3. Through the TCGA portal database, we discovered that CCT3 expression may be subject to many driver genes such as APC, TP53 and KRAS in multiple tumors (Figure 11A). In particular, TP53 mutation status showed a significant correlation with CCT3 expression (Figure 11B). Other than genetic factors, epigenetic factors including DNA methylation, histone modification and non-coding RNA are also critical for gene expression (Moore et al., 2013; Audia and Campbell, 2016; Boulos et al., 2020). We observed the presence of CpG islands in the CCT3 promoter region by the Methprimer database (Supplementary Figure S8A). Furthermore, the DNA methylation levels of CCT3 were lower in tumor tissues than normal tissues (Supplementary Figure S8B). In addition, the SangerBox data showed a significant correlation between CCT3 and the methyltransferases (Supplementary Figure S8C). Histone H3K27ac modification is necessary for enhancer to activate the transcription of target genes (Kang et al., 2021). We found a strong H3K27ac signal in CCT3 promoter region by the WashU database in various tumor cells (Figure 12A). BRD4, the H3K27ac signal reader, was positively relevant with CCT3 expression in gastric cancer (Supplementary Figure S8D). Our ChIP assay confirmed the occupancy of H3K27ac and BRD4 in CCT3 promoter region (Figures 12B,C). More importantly, enhancer inhibitors (JQ1 and I-BET-762) or BRD4 knockdown attenuated CCT3 expression in gastrointestinal tumor cells (Figures 12D,E). It is well known that microRNA can regulate gene expression by depressing the translation of the mRNA or by inducing its degradation. Next, we screened the potential miRNAs for CCT3 by using the StarBase, Targetscan, MiRDB, MiWALK and DIANA database, resulting in six common members (Figures 13A,B). Finally, we predicted the lncRNAs and circRNAs for the above six miRNAs through the StarBase database (Figure 13C). Collectively, we revealed various genetic and epigenetic factors responsible for CCT3 expression.

## Discussion

Molecular chaperone CCT played a central role in tumorigenesis (Narayanan et al., 2016). As one of the significant subunits of CCT, CCT3 regulated the folding process of 7% cytosolic proteins such as VHL, tubulin, actin and cyclin E (Sternlicht et al., 1993; Willison, 2018; Manna et al., 2019). As a chaperon protein, CCT3 was critical for cancer pathogenesis by regulating cell apoptosis, proliferation and energy metabolism. CCT3 has been studied in multiple tumor types. This study wanted to further explore CCT3’s roles through a comprehensive pan-cancer analysis workflow, contributing to revealing the similarity and difference among different tumors. Our study showed a widespread high expression of CCT3 in pan-cancer, especially in 8 cancer types, suggesting that CCT3 may function as an oncogene in tumors. Given its correlation with tumor stages and grades, CCT3 may act as a new diagnostic biomarker. CCT3 has been identified as a prognostic factor in HNSC and HCC (Cui et al., 2015; Wang et al., 2021). Similarly, we affirmed that the tumor patients with high-expressed CCT3 showed poor prognosis, demonstrating its potential as a prognostic biomarker for multiple tumor types.

As an emerging treatment for tumors, immunotherapy has showed significant improvement in the survival time and the quality of life (Esfahani et al., 2020). Extraordinarily, immune checkpoint inhibitors (ICI) have been applicated in a variety of cancers. However, the adverse effects and high cost pose an obstacle to its application. Therefore, screening out the patients who are really suitable for immunotherapy is required (Hou et al., 2021). Owing to the heterogeneity of tumors, single-cell sequencing analysis is becoming popular. Our study observed an abnormal increase of CCT3 expression in multiple immune cells of the tumor microenvironment based on single-cell sequencing data. Moreover, we found a significant negative correlation of CCT3 expression with a variety of immune cells in various carcinomas. Additionally, CCT3 expression was negatively relevant with the immune checkpoint genes expression including CD274, PDCD1 and CTLA4. That means that the tumor patients with high CCT3 expression may not be suitable for immune checkpoint inhibitors, contributing to selecting the exact patients. Considering that CCT3 was relevant with an immunosuppressive tumor microenvironment, we assumed whether CCT3 overexpression lead to immune escape? Indeed, our results found CCT3 expression was negatively correlated with the function of cytotoxic T lymphocyte, B cell receptor signaling pathway and NK cell mediated cytotoxicity. How does CCT3 regulate tumor microenvironment? This may be attributed to CCT3’s key roles in regulating amino acid metabolic process. Amino acid metabolism is essential for driving drug resistance including immunotherapy (Yoo and Han, 2022). In addition to energy generation, amino acid metabolism could also support cancer cells by maintaining redox homeostasis. For example, reactive oxygen species (ROS) produced by amino acid metabolism, are associated with immunosuppression by acting as signaling messengers (Chen et al., 2016). ROS elevated in the tumor microenvironment, could suppress T cell activation, apoptosis, and hyporesponsiveness. Growing evidences suggest that targeting amino acid metabolism is effective in simulating anti-tumor immune response (Ananieva, 2015; Sosnowska et al., 2021). Thus, we hypothesize that CCT3 may regulate amino acid metabolism to inhibit the functions of immune cells in COAD, contributing to immune escape.

We further explored the oncogenic roles of CCT3 in colon cancer by vitro assay. Here, we were the first study to report that targeting CCT3 significantly inhibited colon cancer cells proliferation. GSEA analysis indicated that CCT3 promoted cell growth by means of regulating cell cycle, which was consist with the results in cervical cancer (Dou and Zhang, 2021). Previous studies elucidated that CCT3 promoted tumor cell proliferation by mediating YAP activity (Liu et al., 2019; Shi et al., 2022). Our functional enrichment analysis showed that CCT3 may be oncogenic by regulating multiple pathways such as AMPK and Hippo signaling pathway. All these data implied that CCT3 could be a new molecular target. Of course, more *in vivo* and vitro assays are needed to validate our conclusion. Subsequently, our study screened out sensitive drugs for CCT3 protein by sensitivity analysis and virtual screening. We obtained 5 drugs derived from the FDA-approved clinical trials, which provided a rationale for targeting CCT3. Among them, AT-7519 has showed a therapeutic efficacy for non-small cell lung cancer patients with concurrent chemo-radiotherapy resistance (Liu et al., 2022b). And AT-7529 could be also noticed as potential drugs for HER2-positive breast cancer (Khanjani et al., 2021). PKI-587 enhanced chemosensitivity and radiosensitization of HCC by inhibiting PI3K/AKT/mTOR pathway (Zhang et al., 2019; Xie et al., 2021). Moreover, PKI-587 demonstrated anti-tumor activity in ovarian cancer xenograft models (Langdon et al., 2019). Whether these two drugs play their roles of anti-tumor by targeting CCT3? This needs further experiments to validate. However, there is still no research about the effect of AZD-3147, Kahahide F and LY-3023414 on cancers. The safety and effectiveness of these drugs needs to be further verified by *in vitro* and *in vivo* assays.

Meanwhile, we also investigated the regulatory mechanisms for CCT3 overexpression in tumors. We discovered that CCT3 mutation was seldom. However, many driver genes, TP53 in particular, were responsible for CCT3 overexpression. TP53 depressed tumor growth and promoted cell cycle arrest, apoptosis and senescence in response to diverse forms of cellular stress (Levine and Oren, 2009; Pope et al., 2021). Certainly, how mutant TP53 affected CCT3 expression needs further exploration. Besides, epigenetic factors were indispensable for CCT3 overexpression. The epigenetic factors contain DNA methylation, histone modification and non-coding RNA. Among them, histone acetylation, especially histone three lysine 27 acetylation (H3K27ac), has intrigued more attention. H3K27ac modification is used as the best marker to identify enhancers. Enhancers play important roles in controlling cellular states in human cancers, which provides novel therapeutic targets for cancer treatment (Ye et al., 2021). Unexpectedly, our study shed light on the effect of enhancers on CCT3 expression by a series of *in vitro* assays. Therefore, our research provided another proof for the crucial roles of enhancers in cancers. It is well known that miRNA can regulate gene expression by regulating the translation efficiency of mRNA, as well as its degradation (Guo et al., 2010). While lncRNA or circRNA could influence gene expression by acting as molecular “sponges” (Dai et al., 2015). Therefore, we predicted and constructed the lncRNA/circRNA-miRNA-CCT3 regulatory network. Similarly, Qu et al. discovered that CCT3 was a direct target of miR-223 (Qu et al., 2020). Interestingly, they also found CCT3 could regulate Wnt/β-catenin signaling pathway activity by miR-223. A positive regulatory loop may exist between CCT3 and miR-223 in breast cancer. Among the predicted lncRNA/circRNA, KCNQ1OT1 was involved in the regulation of tumor microenvironment in colon cancer by regulating CD155 expression (Lin et al., 2021b; Liu et al., 2021). Even exosome-derived KCNQ1OT1 could mediate immune escape by regulating PD-L1 ubiquitination in colon cancer (Xian et al., 2021). Of course, specific assays are needed to validate the regulatory relationship between KCNQ1OT1 and CCT3. The regulatory network could not only be used to explain the reasons for CCT3 overexpression, but also provide potential therapeutic targets for tumors. In summary, CCT3 expression was subject to genetic and epigenetic factors in tumors. Our results revealed the oncogenic roles and driving forces of CCT3 in tumors, providing clues for the research of targeting CCT3 in human tumors.

## Conclusion

CCT3 had the potential to be a diagnostic and prognostic biomarker for multiple tumor types. CCT3 expression was relevant with an immunosuppressive tumor microenvironment. CCT3 could be a new molecular target for colon cancer. Both genetic and epigenetic factors were responsible for CCT3 expression in tumors.

## Data Availability

The datasets presented in this study can be found in online repositories. The names of the repository/repositories and accession number(s) can be found in the article/Supplementary Material.
