# Supplementary material for: Insights into the roles and driving forces of CCT3 in human tumors
Source: Front Pharmacol. 2022 Oct 12;13:1005855. doi: 10.3389/fphar.2022.1005855 (PMC9596777; doi:10.3389/fphar.2022.1005855)
Supplement: Supplementary file 1 [file DataSheet1.pdf]

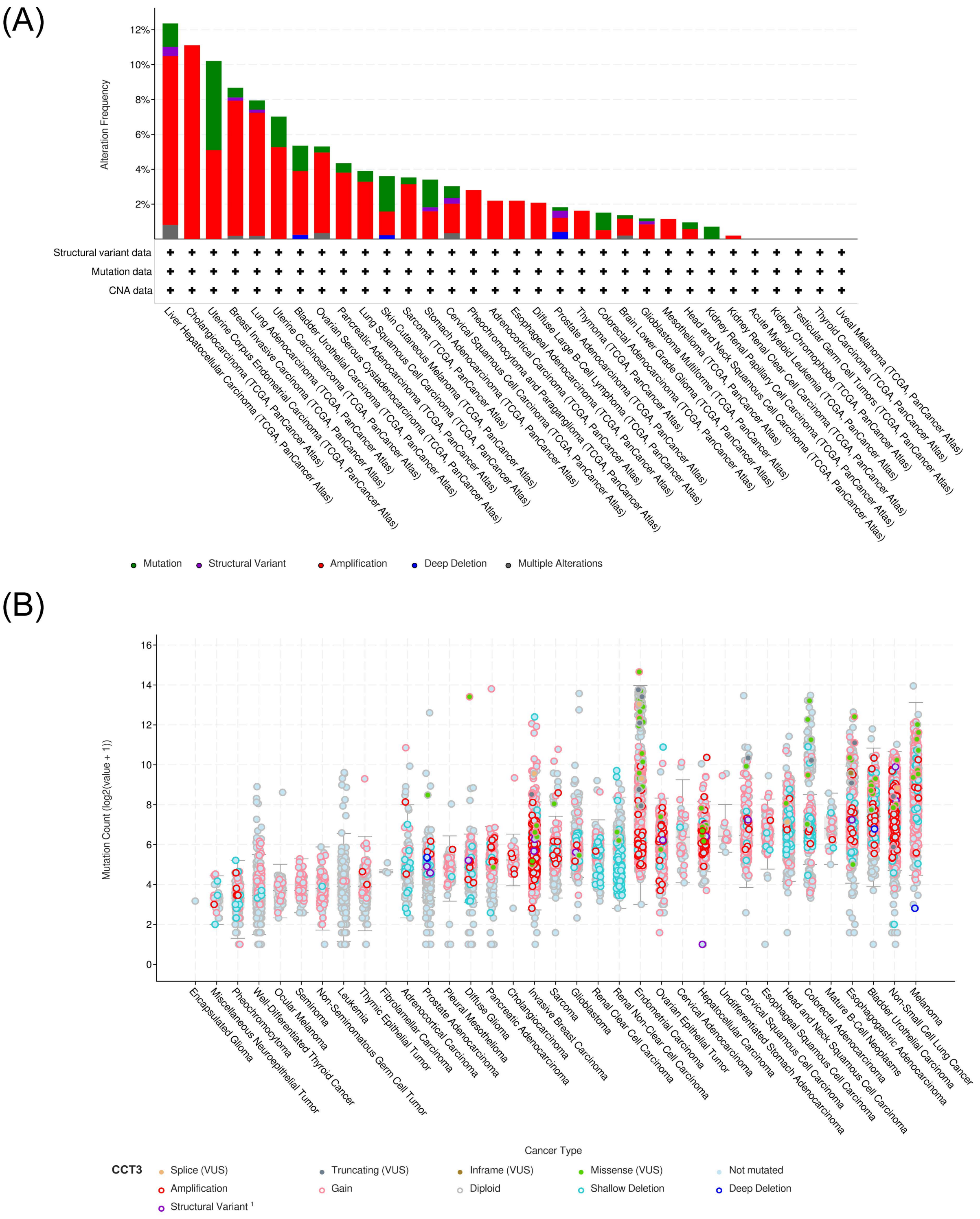

Figure S1. The mutation status of CCT3. (A-B) Mutation frequency and count of CCT3 was explored using the cBioPortal database.

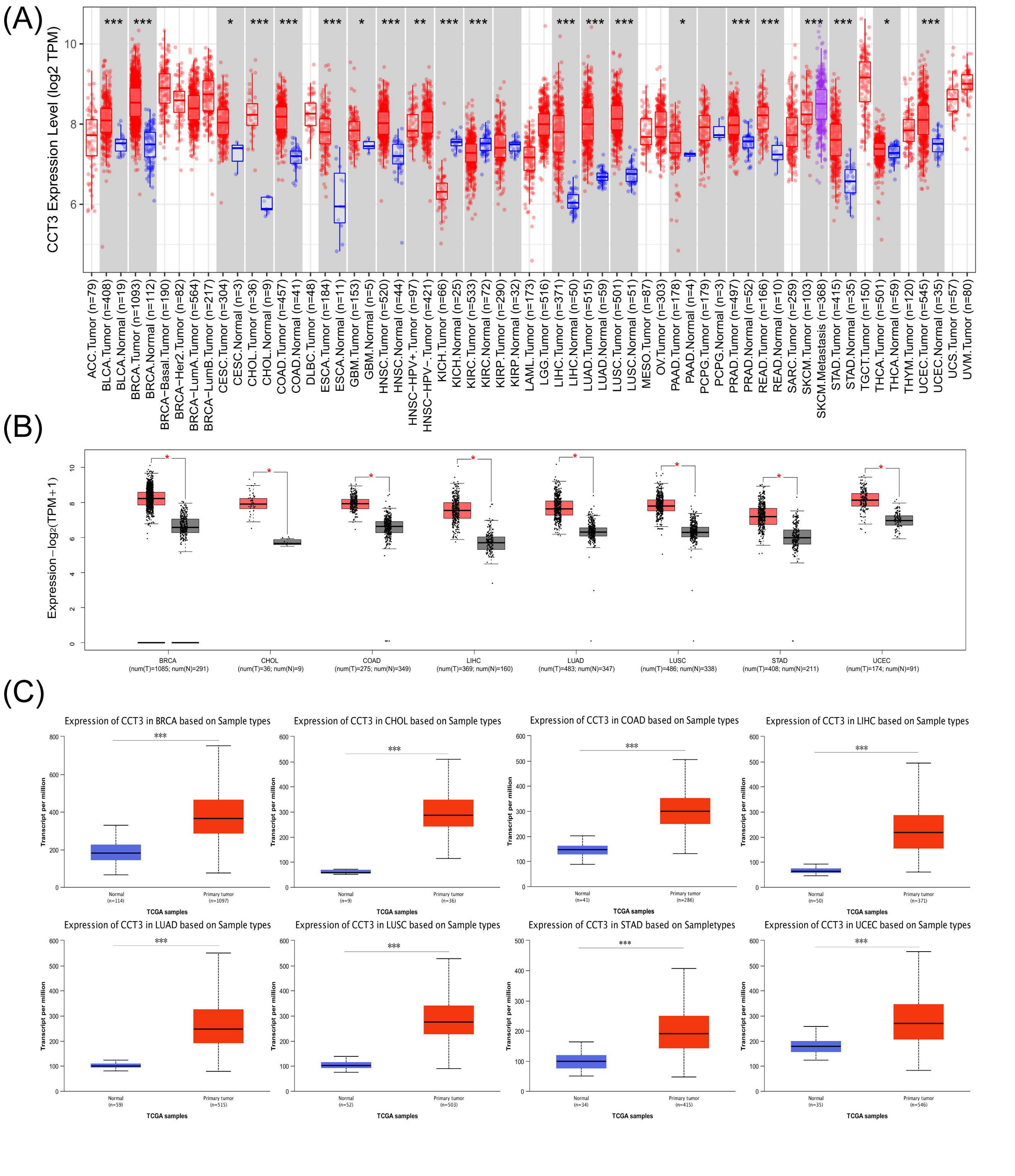

Figure S2. Expression levels of CCT3 in pan-cancer. (A) Differential expression levels of CCT3 in pan-cancer were explored by the TIMER database. (B-C) Differential expression levels of CCT3 in 8 cancer types were explored by GEPIA2 and UALCAN databases.

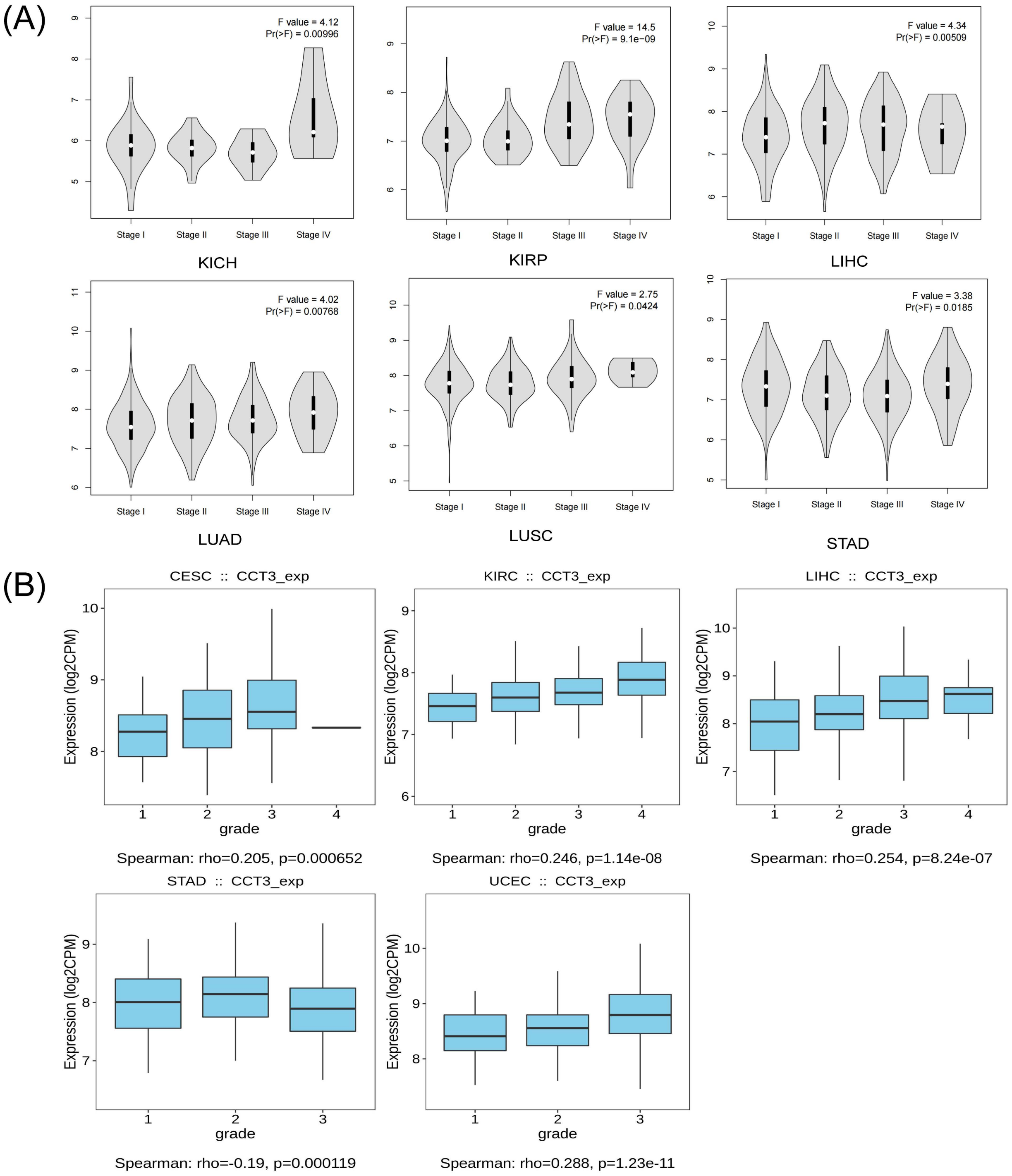

Figure S3. Correction between CCT3 expression and pathological stages, tumor grades in pan-cancer. (A) The expression of CCT3 in different pathological stages was analyzed by the GEPIA2 database. (B) The correction between CCT3 expression and tumor grades was analyzed by the TISIDB database.

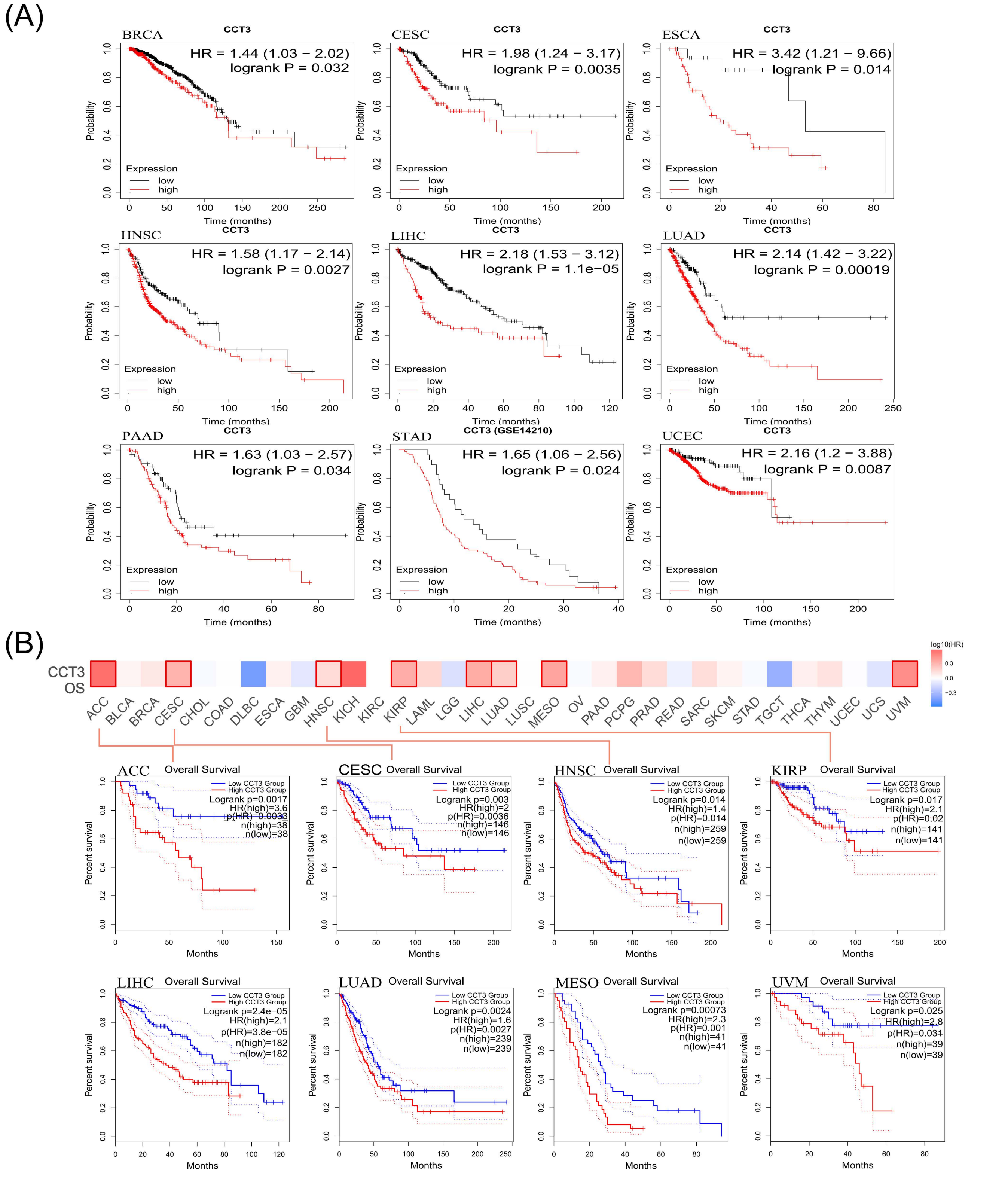

Figure S4. The prognostic value of CCT3 in pan-cancer. (A) The prognostic value of CCT3 for OS was analyzed using the Kaplan-Meier Plotter database. (B) The prognostic value of CCT3 for OS was analyzed by the GEPIA2 database.

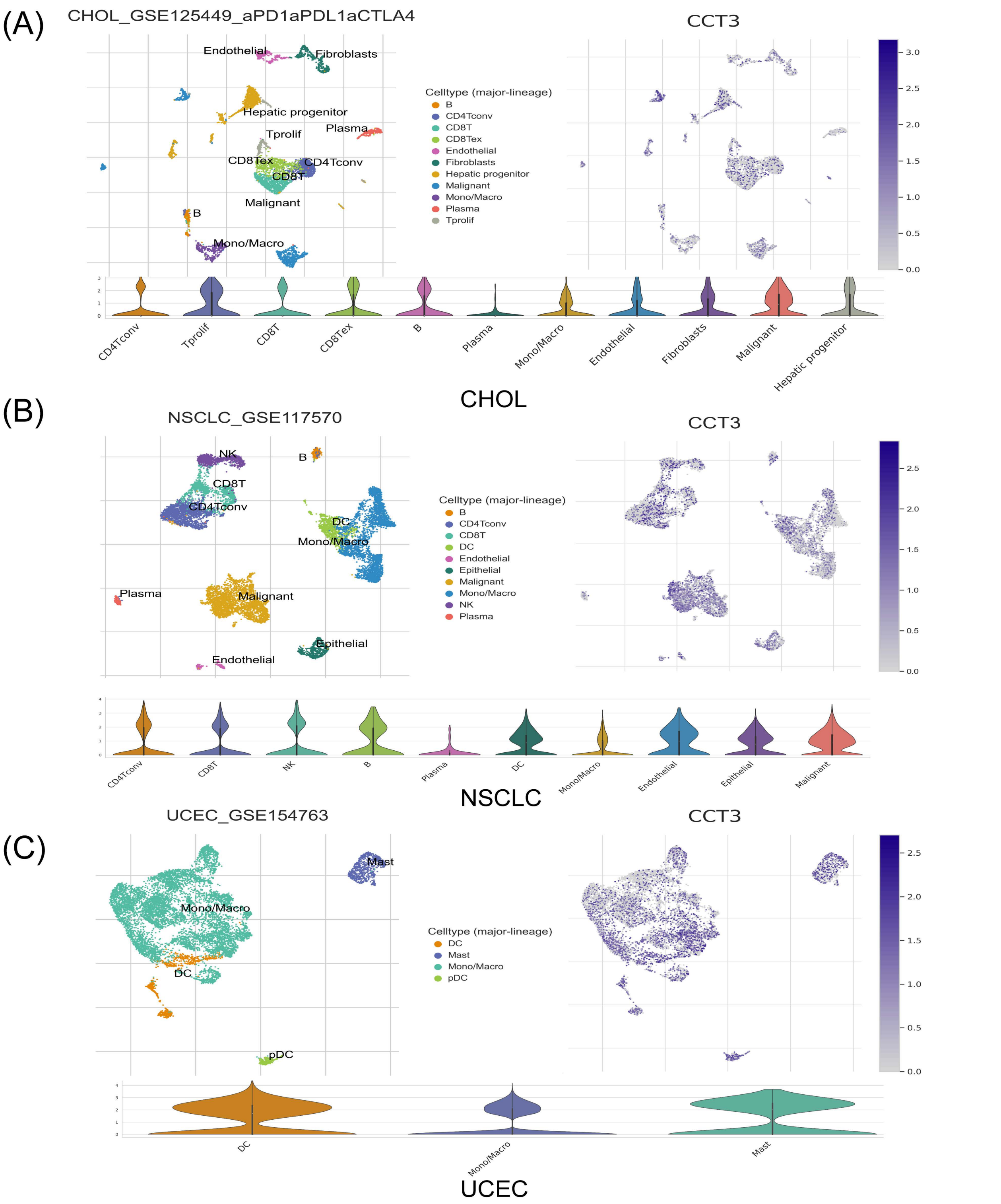

Figure S5. CCT3 expression based on the single-cell sequencing data. (A-C) CCT3 expression in various cell types was investigated by single-cell sequencing data in CHOL, NSCLC and UCEC.

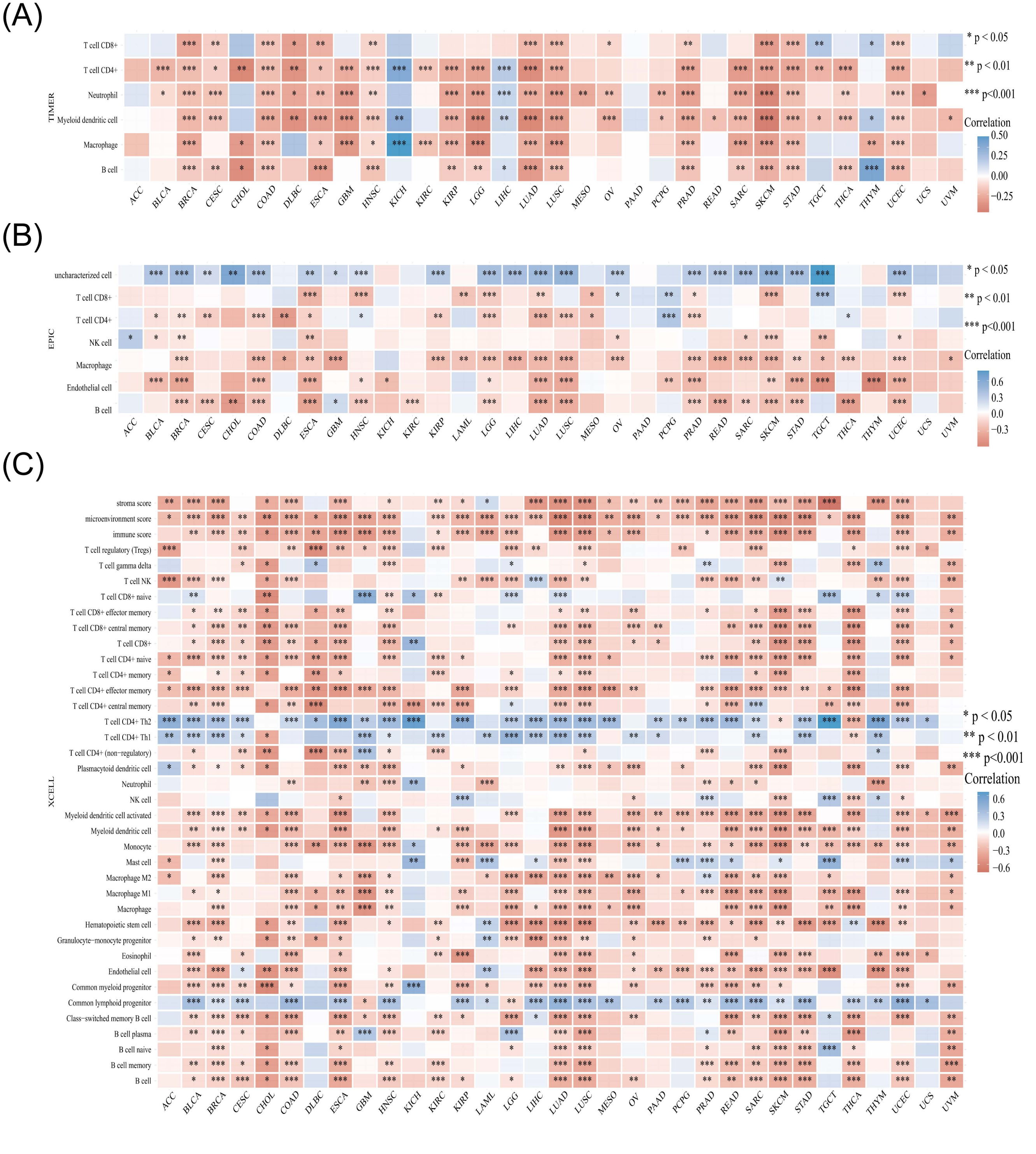

Figure S6. Correlation between CCT3 expression and immune cell infiltration. (A-C) Correlation between CCT3 expression and immune cell infiltration was analyzed in the R package "immunedeconv" using the TIMER, EPIC and xCELL algorithm.

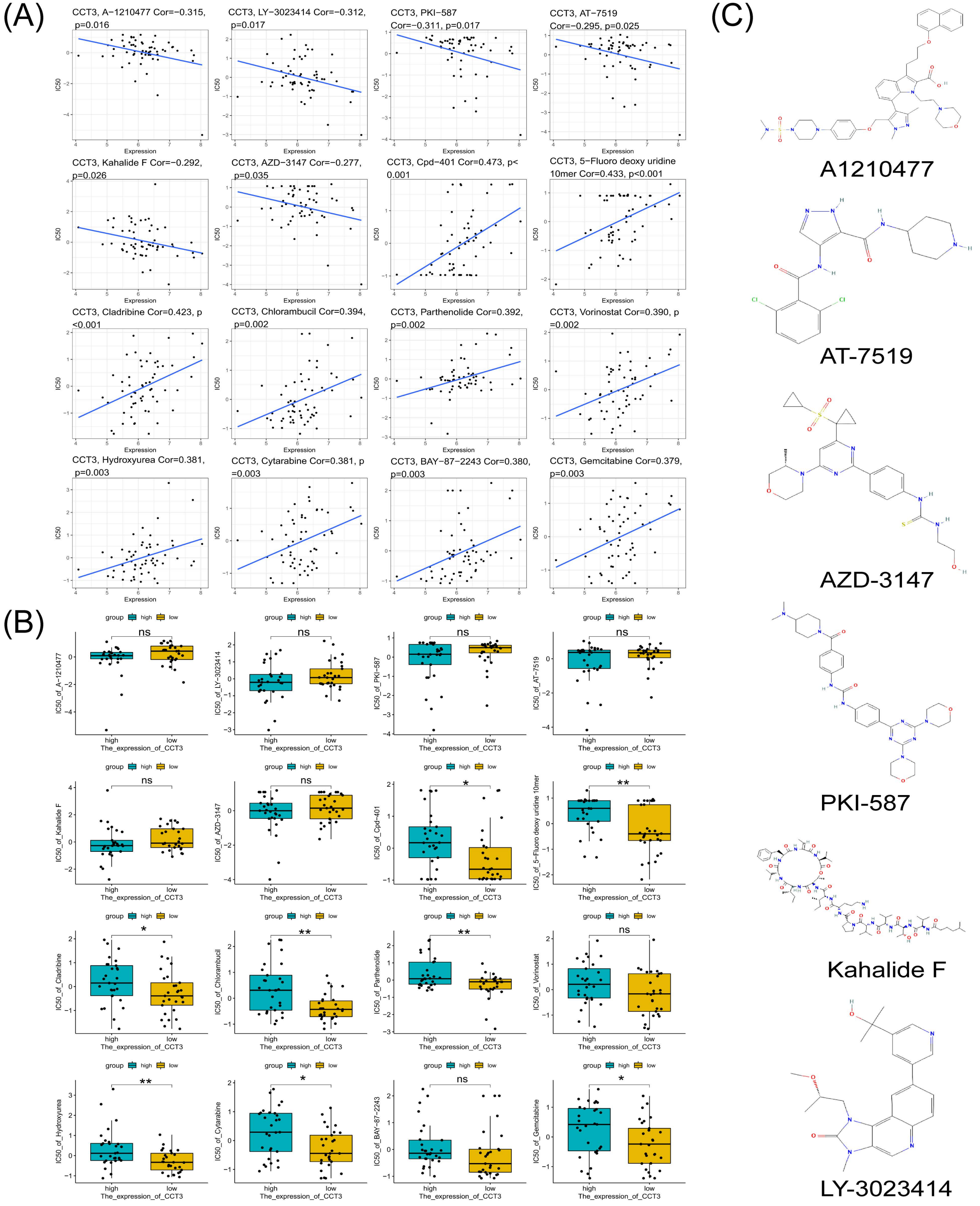

Figure S7. Screening sensitive drugs for CCT3. (A) Scatter plot of the CCT3-sensitive drugs. (B) Boxplot showed the differences between drug sensitivity and CCT3 expression. (C) Compound structure of the 6 CCT3-sensitive drugs.

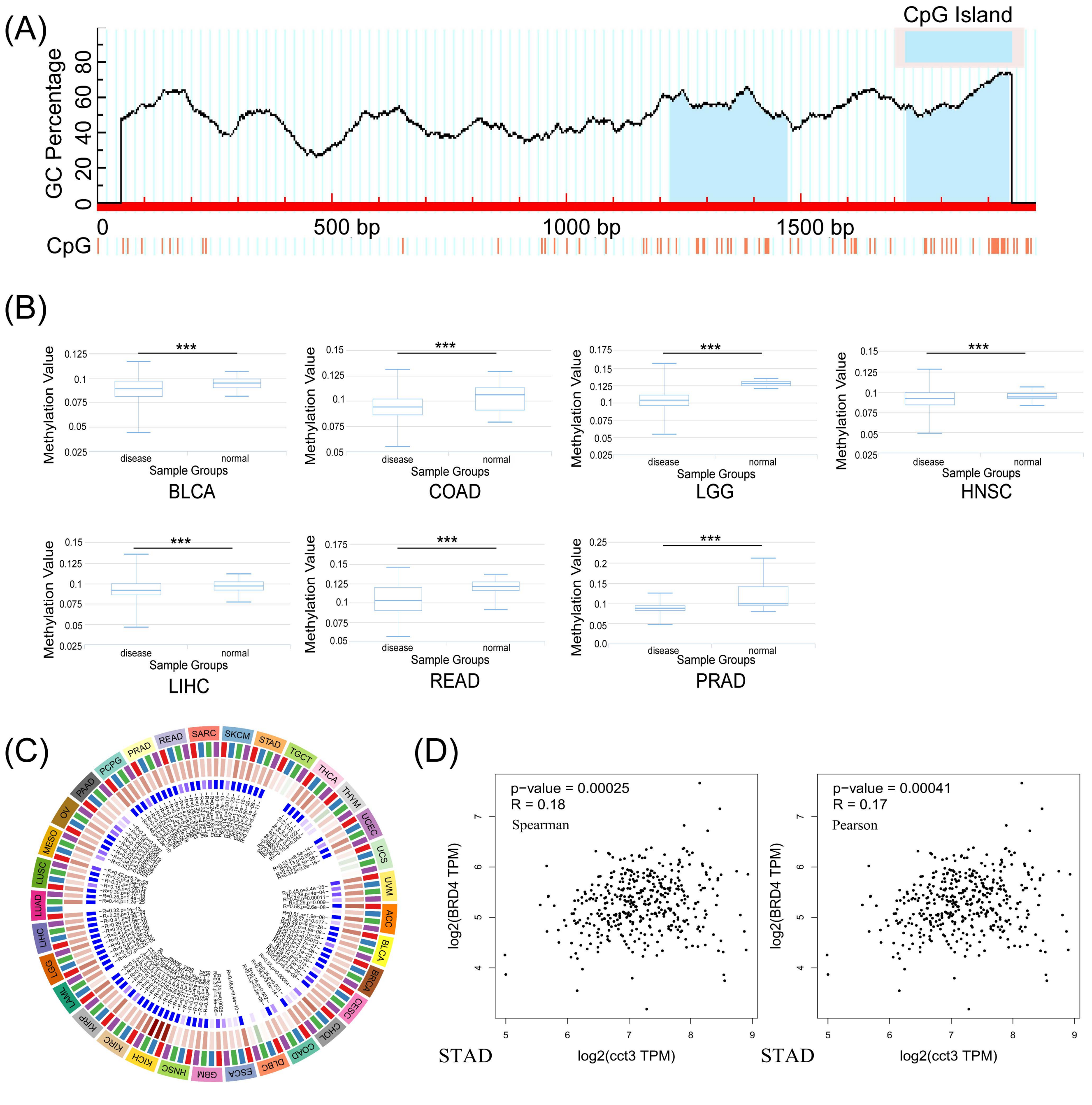

Figure S8. The correlation between DNA methylation and CCT3 expression. (A) CpG islands in the CCT3 promoter region using the Methprimer database. (B) The DNA methylation levels of CCT3 in tumor and normal tissues. (C) The correlation between CCT3 and the methyltransferases was visualized by the Sangerbox database. (D) The correlation between CCT3 and BRD4 expression by GEPIA2 database. *p < 0.05, **p < 0.01, ***p < 0.001.

Supplementary Table 1. List of abbreviations

Abbreviations	Full name
ACC	Adrenocortical carcinoma
BLCA	Bladder Urothelial Carcinoma
BRCA	Breast invasive carcinoma
CESC	Cervical squamous cell carcinoma and endocervical adenocarcinoma
CHOL	Cholangiocarcinoma
COAD	Colon adenocarcinoma
COADREAD	Colon adenocarcinoma/Rectum adenocarcinoma Esophageal carcinoma
DLBC	Lymphoid Neoplasm Diffuse Large B-cell Lymphoma
ESCA	Esophageal carcinoma
GBM	Glioblastoma multiforme
GBMLGG	Glioma
HNSC	Head and Neck squamous cell carcinoma
KICH	Kidney Chromophobe
KIPAN	Pan-kidney cohort (KICH+KIRC+KIRP)
KIRC	Kidney renal clear cell carcinoma
KIRP	Kidney renal papillary cell carcinoma
LAML	Acute Myeloid Leukemia
LGG	Brain Lower Grade Glioma
LIHC	Liver hepatocellular carcinoma
LUAD	Lung adenocarcinoma
LUSC	Lung squamous cell carcinoma
MESO	Mesothelioma
OV	Ovarian serous cystadenocarcinoma
PAAD	Pancreatic adenocarcinoma
PCPG	Pheochromocytoma and Paraganglioma
PRAD	Prostate adenocarcinoma
READ	Rectum adenocarcinoma
SARC	Sarcoma
STAD	Stomach adenocarcinoma
SKCM	Skin Cutaneous Melanoma
STES	Stomach and Esophageal carcinoma
TGCT	Testicular Germ Cell Tumors
THCA	Thyroid carcinoma
THYM	Thymoma
UCEC	Uterine Corpus Endometrial Carcinoma
UCS	Uterine Carcinosarcoma
UVM	Uveal Melanoma
BRD4	bromodomain containing 4
CCT	chaperonin-containing TCP-1
CCT3	chaperonin containing TCP1 subunit 3
CD274	CD274 molecule
CNV	copy number alteration

CTLA4	cytotoxic T-lymphocyte associated protein 4
DFI	disease-free interval
DNMT1	DNA methyltransferase 1
DNMT2	DNA methyltransferase-2
DNMT3A	DNA methyltransferase 3 alpha
DNMT3B	DNA methyltransferase 3 beta
DSS	disease-specific survival
HAVCR2	hepatitis A virus cellular receptor 2
HSF1	heat shock transcription factor 1
HSP	heat shock protein
IHC	Immunohistochemistry
KRAS	KRAS proto-oncogene
LAG3	lymphocyte activating 3
OS	Immunohistochemistry
PDCD1	programmed cell death 1
PDCD1LG2	programmed cell death 1 ligand 2
PD-L1	CD274 molecule
PFI	progression-free interval
STAT3	signal transducer and activator of transcription 3
TP53	tumor protein p53

Supplementary Table 2. CCT3 is ranked from small to large according to the P-values among the upregulated differential genes (Fold Change>1.5, p value<0.05) in 8 cancer types. Sum: Total number of upregulated differential genes.

Tumor	Gene	Sum	Rank	baseMean	log2FoldChange	pvalue	padj
BRCA	CCT3	5126	235	26060.54397	1.172963103	2.16E-87	7.34E-86
CHOL	CCT3	4961	2320	13592.61368	0.84334095	2.38E-06	9.10E-06
COAD	CCT3	4422	254	12652.71701	0.987228498	1.58E-53	6.61E-52
LIHC	CCT3	5303	348	15499.33832	1.182954261	3.23E-35	1.44E-33
LUAD	CCT3	5335	293	15148.6417	1.3742316	3.85E-59	1.51E-57
LUSC	CCT3	6192	462	18791.91407	1.385296125	7.44E-79	2.20E-77
STAD	CCT3	3505	1213	14480.8471	0.59908591	3.86E-10	2.80E-09
UCEC	CCT3	5630	800	11996.24647	0.910361266	4.35E-25	5.20E-24

Supplementary Table 3

Energy values docking simulation of CCT3 with compounds.

Protein	Compounds	Estimated Free Energy of Binding
CCT3	A1210477	+0.43 kcal/mol
CCT3	AT-7519	-4.64 kcal/mol
CCT3	AZD-3147	-2.24 kcal/mol
CCT3	Kahalide F	-3.35 kcal/mol
CCT3	LY-3023414	-4.16 kcal/mol
CCT3	PKI-587	-5.28 kcal/mol
